# Epidermal MHC-II-mediated NK cell recruitment triggers keratinocyte pyroptosis, facilitating pathogenesis of psoriasis

**DOI:** 10.1038/s12276-026-01717-z

**Published:** 2026-05-08

**Authors:** Xiaoqing Yi, Pian Yu, Jiayi Wang, Chi Fang, Sihui Ma, Kaixuan Li, Rongxuan Yan, Guanming Wang, Yihui Chen, Chao Chen, Detian Zhang, Yehong Kuang, Wu Zhu, Jie Li, Guoqiang Zhang, Tuo Deng, Xiang Chen, Cong Peng

**Affiliations:** 1 The Department of Dermatology, Xiangya Hospital, Central South University, Xiangya, China; 2 Hunan Key Laboratory of Skin Cancer and Psoriasis, Hunan Engineering Research Center of Skin Health and Disease, Xiangya Hospital, Xiangya, China; 3 Furong Laboratory, Changsha, China; 4 National Engineering Research Center of Personalized Diagnostic and Therapeutic Technology, Xiangya, China; 5 National Clinical Research Center for Geriatric Diseases, Xiangya Hospital, Xiangya, China; 6 Department of Dermatology, The First Hospital of Hebei Medical University, Shijiazhuang, China; 7 National Clinical Research Center for Metabolic Diseases, Department of Metabolism and Endocrinology, The Second Xiangya Hospital of Central South University, Changsha, China; 8 Key Laboratory of Diabetes Immunology, Ministry of Education, Changsha, China; 9 Metabolic Syndrome Research Center, Clinical Immunology Center, Central South University, Changsha, China

**Keywords:** Cell death and immune response, Psoriasis

## Abstract

Psoriasis is a chronic inflammatory disease characterized by dysregulated interactions between keratinocytes (KCs) and immune cells. However, the details of KCs orchestrating the immune cell infiltration, particularly for natural killer (NK) cells, remain unclear. Here NK cell infiltration is significantly increased in psoriatic skin lesions, and the application of anti-MHC-II treatment or knockout of H2-Ab1 in the epidermis dramatically reduces IMQ-mediated psoriatic dermatitis as well as the infiltration of NK cells through CXCL10 mediated by the ERK–CREB axis. Spatial transcriptomics reveal that NK cells coexist with KCs, driven by enhanced CXCL signaling, and epidermal H2-Ab1 deletion suppresses KC–NK cell communication. NK cells release granzyme B, inducing pyroptosis in adjacent GSDME-expressing KCs, contributing to the inflammatory response. NK cell depletion or Gsdme knockout reduces pyroptosis and alleviates psoriasis-like dermatitis. Multicolor immunohistochemistry confirms that epidermal MHC-II expression in psoriatic lesions correlates positively with NK cell, granzyme B and cleaved-GSDME levels. This study reveals that epidermal MHC-II attracts NK cells, triggering KC pyroptosis and remodeling the immune microenvironment in psoriasis, offering novel insights into its pathogenesis.

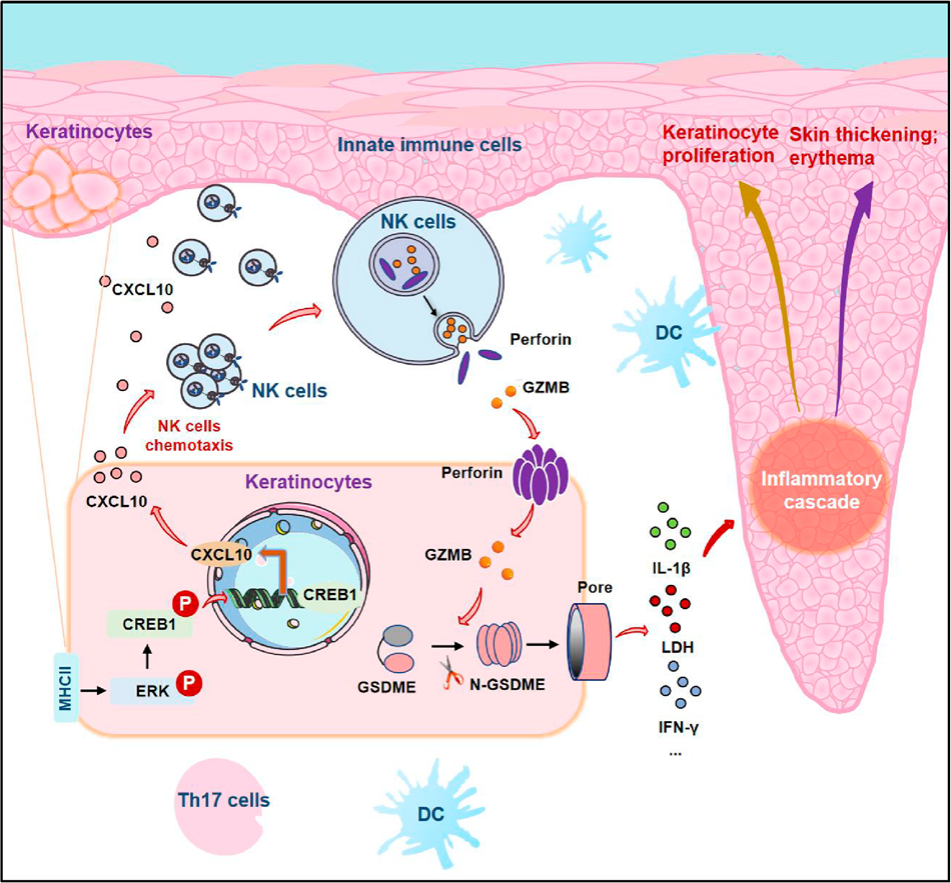

## Epidermal MHC-II drives NK cell infiltration and GSDME-mediated pyroptosis in psoriasis lesions

Psoriasis is a chronic inflammatory skin condition driven by complex interactions between immune cells and keratinocytes (KCs). This study explores the role of MHC-II molecules in KCs, revealing their impact on natural killer (NK) cell function and pyroptosis. Using single-cell and spatial transcriptomics, the authors demonstrate that MHC-II expression in KCs enhances NK cell recruitment and activation, leading to increased pyroptosis in psoriatic lesions. The study utilized human samples and mouse models, including KC-specific H2-Ab1 knockout mice, to investigate these interactions. Results showed that blocking MHC-II,NK cells or GSDME reduced psoriasis severity, highlighting the role of NK cell-derived granzyme B in activating GSDME-mediated pyroptosis. These findings suggest that targeting MHC-II, NK cells or pyroptosis could offer new therapeutic strategies for psoriasis, with further research warranted to validate these findings in clinical settings.

This summary was initially drafted using artificial intelligence, then revised and fact-checked by the author.

## Introduction

Psoriasis, a complex inflammatory skin disease with diverse clinical manifestations, is caused by various etiologies involving genetic, immunological and environmental factors^1–3^ and is characterized by erythema covered in silvery scales with thickening of the skin^1^. Abnormal immune responses as well as dysregulated keratinocyte (KC) proliferation and differentiation are the central events in the progression of psoriasis^4^. Although KCs form the outermost layer of the skin, they also contribute to the pathogenesis of psoriasis by interacting with other cell types, including T cells, dendritic cells (DCs) and so on, through the secretion of proinflammatory cytokines or chemokines, which are involved in the initiation, amplification and maintenance of psoriasis^5^.

The interaction between DCs and T_H_17 cells plays a key role in the pathogenesis of psoriasis^6^. The antimicrobial peptide LL-37 produced by KCs activates DCs when released with self-DNA. Once activated, DCs stimulate the release of cytokines such as IL-23 and promote the differentiation of naive CD4^+^ T cells into T_H_17 cells. T_H_17 cells are well known for their ability to produce pathogenic IL-17A, which acts as a potent promoter of KC proliferation and differentiation, fueling the inflammatory cascades and leading to thickened, scaly and erythematous lesions that are hallmarks of the disease^7,8^. Therefore, the IL-23–IL-17A axis is considered as the primary inflammatory pathway driving the pathogenesis of psoriasis, which promotes the activation and proliferation of immune cells, leading to a chronic proinflammatory state in psoriasis^9^. Topical application of imiquimod (IMQ) and subcutaneous injection of IL-23 could rapidly induce psoriasis-like dermatitis, critically dependent on the activation of the IL-23–IL-17 axis^10^. Therefore, IMQ- and IL-23-induced models have been widely used in preclinical research to investigate the molecular and cellular mechanisms of psoriasis^11^.

KCs are not only involved in triggering psoriasis development but also act as the primary executors of the disease^7^, which could induce the expression of various psoriasis-related inflammatory factors such as S100A8, S100A9 and so on, when stimulated by cytokines such as IL-17A^12^. As a result, KCs play a critical role in sustaining the chronic inflammation in psoriasis. By destructing the interactions between cytokines and KCs, the inflammatory response can be reversed, resulting in improved outcomes for patients with psoriasis^13^. Therefore, identifying novel molecules for targeted therapy by regulating KC functions may lead to more successful outcomes for psoriasis therapy.

MHC molecules, also known as human leukocyte antigen (HLA) in humans and H-2 complex in mice, are polymorphic cell surface proteins that present antigenic peptides to T cells by forming the macromolecular substance, which is necessary for T cell recognition and activation, as well as the subsequent immune response^14^. MHC-II is expressed on the DCs, macrophages, B cells and several types of non-professional antigen-presenting cell (APC) such as epithelial, vascular and connective tissue cells in response to inflammatory signals including IFN-γ^15^. MHC-II molecules play critical roles in a variety of immunological disorders, including cancer, infection and autoimmune diseases^16,17^. Although studies have shown that certain genes located within the HLA class II region, such as specific alleles of the *DRB1* genes, are positively associated with susceptibility to psoriasis^18^, the role of MHC-II in psoriasis has not been fully characterized.

In this study, we uncovered the role of MHC-II molecules expressed in KCs in regulating NK cell function and inducing pyroptosis during psoriasis inflammatory responses using single-cell and spatial transcriptomics, which provided an innovative perspective on the mechanism of psoriasis pathogenesis as well as the potential strategies for psoriasis therapy by targeting MHC-II, NK cells or pyroptosis.

## Materials and methods

### Human samples

A total of 39 patients with psoriasis who underwent skin biopsy for pathological examination and were diagnosed with psoriasis vulgaris by experienced dermatologists were recruited from the outpatient clinic of Xiangya Hospital, Central South University. Sex- and age-matched healthy volunteers with nevus excision were recruited, and skin specimens were taken at least 2 mm away from the edge of the nevus. The information of patients with psoriasis (Pso) and healthy controls (HCs) is presented in Supplementary Table 1. Written informed consent was obtained, and the acquisition of human skin samples was approved by the local ethics institutional review board (Xiangya Hospital, IRB-202308636).

### Mice

*H2-Ab1*^*fl/fl*^ (C57BL/6Smoc-*H2-Ab1*^*em1(flox)Smoc*^) and OT-II transgenic mice (C57BL/6Smoc-*Igs2*^*em1(CD2-TCRa(OT-II)-CD2-TCRb(OT-II))Smoc*^) were obtained from Tuo Deng’s Laboratory at the Second Xiang Hospital in Central South University^19^. Krt14-CreERT2 (K14-Cre, C57BL/6Smoc-*Krt14*^*em2(CreERT2-Wpre-polyA)Smoc*^) mice were purchased from Shanghai Biomodel Organism Science and Technology Development Co. *H2-Ab1*^*fl/fl*^ mice were crossed with K14-Cre mice to create KC-specific H2-Ab1 knockout (KO) mice (*K14.H2-Ab1*^*fl/fl*^). *Gsdme*^−/−^ (*Gsdme* KO, C57BL/6Smoc-*Gsdme*^*em1Smoc*^) mice and wild-type (WT) littermates were obtained from Yanfang Xu’s laboratory at Fujian Medical University^20^. All the mice were kept in a specific pathogen-free environment and utilized under the approval of the Ethics Committee of Central South University (#CSU-2022-01-0424). For the in vivo study, the C57BL/6 mice were intraperitoneally injected with 200 μg InVivoMab anti-mouse MHC Class II (I-A/I-E) (BioXcell) or 200 μg anti-mouse NK1.1-InVivo (Selleck) or control IgG (BioXcell; Selleck). BALB/c mice were intraperitoneally injected with Z-DEVD-FMK at a dose of 200 mg/kg (Selleck). Then, 2.5 nmol small interfering RNA (siRNA) targeting Gsdme mixed with emulsion matrix was topically applied to the dorsal skin of mice every other day for 7 days as per the previous recommendations^21^. Mouse strain selection was experiment-oriented: C57BL/6 for genetic models and antibody compatibility, and BALB/c for pharmacological profiling. Dorsal skin was consistently used in GSDME-related studies to ensure comparability and sufficient tissue availability, whereas the ear model was applied for IL-23 stimulation based on established methodology^22^. Finally, mice were euthanized, and the skin tissues were removed for the following hematoxylin and eosin (H&E) staining, flow cytometry, enzyme-linked immunosorbent assay (ELISA), RNA extraction or western blot analysis.

### Cell culture and isolation

HEK-293T, HaCaTs, and NK92 cells were purchased from ATCC. HEK-293T and HaCaTs were maintained in Dulbecco’s modified Eagle medium or 1640 medium plus 10% fetal bovine serum. NK92 was preserved in lymphocyte culture medium supplemented with 10 ng/ml IL-2. Primary KCs were isolated from the skin tissues of infant mice, and details of the sample processing are described in a previous publication^23^. The isolated primary KCs from the epidermis were preserved in the KGM-Gold BulletKit (Lonza). Naive CD4^+^ T cells and NK cells were prepared from the spleen using a CD4^+^CD62L^+^ T cell isolation kit (Miltenyi) or NK cell isolation kit (Miltenyi) following the manufacturer’s instructions.

### Cell co-culture

For KC/T cell co-culture, KCs seeded in the 12-well plate were treated with 250 ng/ml IFN-γ (BioLegend) or not for 24 h, then were incubated with 10 µg/ml OVA (MCE) or not for 2 h at 37 °C in a 5% CO_2_ incubator. The KC growth medium was removed, and the naive CD4^+^ T cells were added and co-cultured in the XVIVO-15 medium (Lonza). Naive CD4^+^ T cells were magnetically sorted from the spleen of OT-II mice. Flow analysis of CD25 and CD69 was used to assess the activation level of naive CD4^+^ T cells. Naive CD4^+^ T cells were prestained 0.5 μM Carboxyfluorescein succinimidyl ester (CFSE; eBioscience) for 15 min at 37 °C, then co-cultured with IFN-γ-treated, OVA-incubated KCs for 3 days. The dilution of CFSE was detected by flow cytometry to evaluate T cell proliferation.

For KC/NK cell co-culture, KCs or HaCaT cells were seeded in the lower chamber of a 12-well plate and stimulated with 100 ng/ml IL-17A (BioLegend) for 24 h. Then, NK cells (NK92) were put into the upper chamber. The co-culture of KC/NK cells was maintained in the XVIVO-15 medium for 24 h. The migration rate is assessed by the number of NK cells (NK92) chemoattracted to the lower well divided by the total NK cells (NK92). KCs co-cultured with NK cells were observed for the cell morphology in the phase-contrast microscope. The cell death levels were measured by LDH-Glo Cytotoxicity Assay (Promega) or propidium iodide (PI; Sigma). Cell lysate was collected to assess GSDME levels by western blot.

### Flow cytometry

Single-cell suspensions of skin were prepared by cutting the skin tissue into small pieces and shaking in 5 ml Dulbecco’s modified Eagle medium plus 2 mg/ml collagenase type IV and 100 μg/ml deoxyribonuclease at 37 °C for 90 min to allow adequate digestion, then 10% fetal bovine serum medium was used to terminate the enzyme activity and the digested tissues were passed through 70-μm cell strainers. The spleen was mashed with a syringe and washed through 40-μm cell strainers with PBS. Single-cell suspensions obtained from the spleens and skin tissues were centrifuged at 500*g* for 5 min, and the cell pellets were labeled with fluorescence antibodies as previously described^24^. The flow antibodies were purchased from BioLegend and listed as follows: APC/Cyanine7 anti-mouse CD45; APC anti-mouse CD3; PE/Dazzle 594 anti-mouse NK1.1; FITC anti-human/mouse Granzyme B recombinant; Brilliant Violet 421 anti-mouse CD4; PE anti-mouse IL-17A; FITC anti-mouse I-A/I-E. The flow-gating strategies for cells isolated from the spleen and skin are shown in Supplementary Fig. 1.

### Western blot

Skin or cell samples were pretreated with RIPA lysis buffer containing protease inhibitor cocktails and phosphatase inhibitors (bimake). The concentration of proteins was further determined by the BCA assay kit. Proteins were first separated by SDS–PAGE gels and transferred to polyvinylidene fluoride membranes, then incubated with anti-MHC Class II antibody (ab139365), H2-I/Abβ (25-9-3)(sc-52537), GAPDH monoclonal antibody (60004-1-Ig), β-actin mouse monoclonal antibody (66009-1-Ig), rabbit monoclonal [EPR19859] to DFNA5/GSDME-N-terminal (ab215191), phospho-p44/42 MAPK (Erk1/2) (Thr202/Tyr204) (D13.14.4E) XP rabbit mAb (#4370), phospho-CREB (Ser133) (87G3) rabbit mAb (#9198), p-p38 MAPK antibody (E-1) (sc-166182) and phospho-JNK (Tyr185) recombinant antibody (80024-1-RR) after blocking with 5% bovine serum albumin. Blots were captured after Enhanced Chemiluminescence (ECL) development on a gel image analysis (Bio-Rad).

### Quantitative real-time PCR (rtPCR)

Skin or cell samples were treated with MagZol reagent (Magen) for RNA extraction. RNA was quantified and reversed into cDNA using HiScript II Q RT SuperMix for qPCR (+gDNA wiper). Actb/ACTB was used as a reference gene expression for calculating the mRNA levels of the target gene. Primer sequences were obtained from Sangon Biotechnology and are listed in Supplementary Table 2.

### ELISA

The skin tissue was weighed and minced in PBS containing protease inhibitors at a 1:9 (weight/volume) ratio. After centrifugation of the homogenate at 5,000*g* for 10 min, the supernatant was collected and analysed for HMGB1 or IL-1β levels using mouse ELISA kits (MEIMIAN) according to the manufacturer’s instructions.

### Histology

Human or mouse skin tissues were fixed in paraformaldehyde, embedded in paraffin and sectioned into 5-μm-thick slides. These sections were used for H&E staining to assess histological features or were subjected to immunohistochemistry with an anti-MHC class II antibody (ab180779) to evaluate MHC-II expression in the epidermis of human skin samples. The immunostaining images were captured under a microscope and quantified by Image Pro-Plus. For multicolor immunohistochemistry, we performed four-color multiplex immunohistochemistry using the Opal 4-color IHC Kit as the manual indicated (AFIHC025). Skin sections from ten patients with psoriasis and ten HCs were respectively stained with anti-CD56 (sc-7326), anti-granzyme B (sc-8022), anti-MHC class II (ab55152) and anti-cleaved Gasdermin E (#55879) antibodies. Opal 690 (MHC-II), Opal 650 (CD56), Opal 520 (GZMB), and Opal 570 (N-GSDME) dyes were used, with DAPI applied as a nuclear counterstain. High-magnification areas in each section were then examined, and the number of MHC-II^+^, CD56^+^, GZMB and cleaved-GSDME^+^ cells per high power field (HPF) was measured by ImageJ.

### Transfection

HLA-DQB1 or CREB1 knockdown in HaCaTs was accomplished with the plasmid purchased from GeneChem with a recombinant lentiviral vector GV248. The lentiviral particles were used to infect HaCaTs with 10 μg/ml polybrene, followed by the selection of 2 μg/ml puromycin. siRNA sequences were designed as required to target mouse Gsdme from RiboBio. Lipo 2000 (Thermo Fisher) was pre-incubated with the Granzyme B/GZMB Protein (TOPSCIENCE) or siRNA for 10 min at room temperature, and then the mixture was added to HaCaTs or primary KCs seeded in the plates to facilitate transfection.

### ChIP assay

The chromatin immunoprecipitation (ChIP) assay was performed using a CREB (48H2) rabbit mAb (CST) to enrich protein/DNA complexes in HaCaTs after cross-linking, lysis and DNA breakage following the manufacturer’s instructions from the SimpleChIP Enzymatic Chromatin IP Kit (CST). Then, the immunoprecipitation reactions in the promoter regions of CXCL10 were verified using specific primer pairs.

### scRNA-seq

The ear skin tissues were washed with Dulbecco’s PBS and soaked in the tissue storage, then transferred to the BGI Qingdao Research Institute. The skin tissues were cut into pieces and enzymatically digested with the Tumour Dissociation Kit (Miltenyi Biotec). After cell filtration and red blood cell lysis, single cells were resuspended in PBS and used to construct barcoded single-cell RNA sequencing (scRNA-seq) libraries on the DNBelab C4 platform. After cDNA production and qualification, sequencing was performed for all the libraries by the DIPSEQ T7 sequencing platform. After obtaining and processing the sequencing data, the gene expression matrix was imported into the Seurat package for further standard quality-control pipeline and analysis workflow.

### Spatial transcriptomics

Mouse skin samples were embedded with optimal cutting temperature compound, then transferred to the Singleron Technology Company for spatial transcriptomics. Frozen tissues were cryosectioned for 10-μm slides. One section was stained with H&E and captured for image under a microscope, aiming to map subsequent gene-expression patterns. Purified RNA from sections was measured for RNA integrity. Samples prepared for sequencing should reach the quality criteria with the RNA Integrity Number (RIN) >4. The H&E-stained section was selected for the proper region and hybridized with probe pairs, then transferred onto a Visium Spatial Gene Expression Slide via the CytAssist system for library construction. Libraries with standard Quality Control (QC) values were sequenced on the Illumina NovaSeq 6000 platform. FASTQ files were aligned, and reads were grouped using the SpaceRanger pipeline version 1.2.2 after sequencing. The final matrix was processed with the R package Seurat v4.0 for the downstream analysis.

### Statistical analysis

All data were present in the form of mean ± standard deviation (s.d.), and statistical analyses were performed in SPSS. The statistical significance was determined by a two-tailed Student’s *t*-test or one-way analysis of variance (ANOVA) with Tukey’s multiple comparisons test. The Anderson–Darling test was used for testing the normal distribution. Spearman’s rank correlation coefficients were calculated to assess the strength of correlations in count data. A *P* value below 0.05 was considered statistically significant.

## Results

### MHC-II is highly expressed in psoriatic skin lesions

**Fig. 1:**
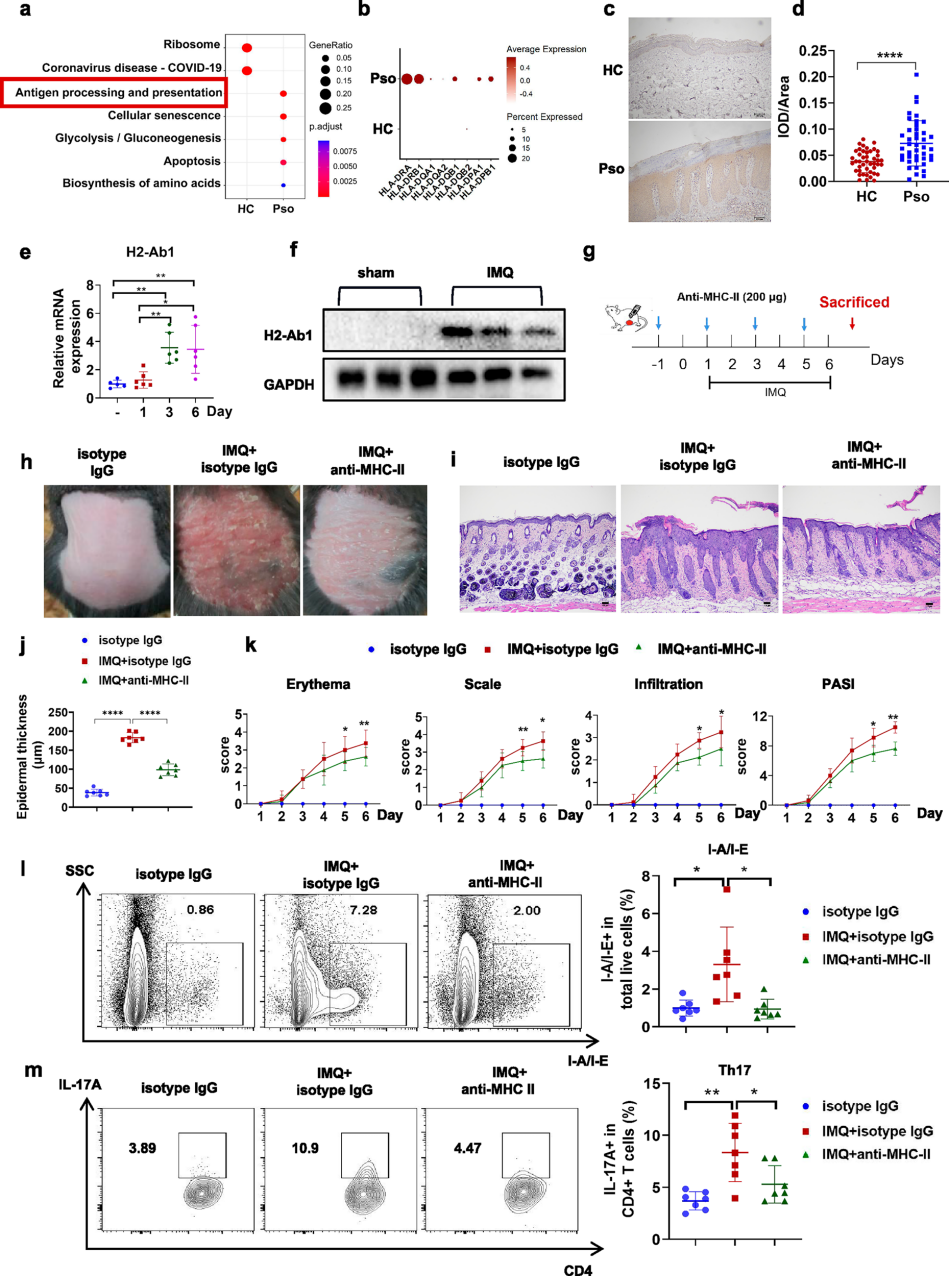
MHC-II participates in the pathogenesis of psoriasis. **a** Enriched KEGG pathways in epidermal tissues from patients with psoriasis (Pso) and HCs based on scRNA-seq analysis. **b** Dot plot showing the increased expression of MHC-II genes in the KC cluster of the patients with psoriasis. **c**, **d** Representative image of immunohistochemical staining and statistical analysis of MHC-II expression in the epidermis of healthy volunteers (HC, *n* = 39) and patients with psoriasis (Pso, *n* = 39). Scale bars, 100 μm. **e** H2-Ab1 mRNA expression in IMQ-treated epidermal tissues. *n* = 6. **f** Immunoblot for the H2-Ab1 level in IMQ-treated epidermis lysate. *n* = 3. **g** Schematic diagram of anti-MHC-II intraperitoneal administration (200 μg) in IMQ-treated mice. **h**–**k** Phenotypic presentation (**h**) and H&E staining (**i**) as well as statistical analysis of the epidermal thickness (**j**) and PASI scores (**k**) of back skin from control or IMQ-treated mice injected with isotype IgG or anti-MHC-II. Scale bars, 100 μm, *n* = 7. **l**, **m** Flow cytometric analysis of the I-A/I-E percentage gated on the total live cells (**l**) or T_H_17 cell percentage gated on the CD4^+^ T cells (**m**) in the skin as indicated. *n* = 7. Data are shown as the mean ± s.d. *P* values were determined using one-way ANOVA with Tukey’s multiple comparisons test or two-tailed Student’s *t*-test. *P < 0.05, **P < 0.01, ***P < 0.001, ****P < 0.0001.

First, our scRNA-seq analysis of epidermal samples from patients with psoriasis and HCs showed an enrichment of the antigen presentation pathway in psoriasis (Fig. 1a). Focusing on the major KC compartment (Supplementary Fig. 2a, b), we identified a significant upregulation of multiple MHC-II gene loci in psoriatic lesions (Fig. 1b). These genes exhibited distinct expression patterns across KC subpopulations, with the strongest enrichment observed in basal KCs (Supplementary Fig. 2c). MHC-II expression was also shown to be increased in the epidermis of patients with psoriasis by immunochemistry (Fig. 1c, d) and demonstrated good predictive performance for psoriasis diagnosis, with an area under the curve (AUC) value greater than 0.7 (Supplementary Fig. 2d). Meanwhile, the mRNA and protein levels of the classical murine MHC-II specific epitope H2-Ab1 were also increased in the epidermis (Fig. 1e, f) and whole skin tissues (Supplementary Fig. 2e–g) after IMQ topical application. These results suggest that MHC-II, particularly when expressed in the epidermis, may play a critical role in psoriasis.

To further investigate the role of MHC-II, we blocked its function by administering an anti-MHC-II antibody and conducted experiments using the IMQ-induced psoriasis-like mouse model (Fig. 1g). Flow cytometry analysis of splenocytes indicated that MHC-II (I-A/I-E) expression was successfully blocked (Supplementary Fig. 2h). Anti-MHC-II treatment significantly reduced psoriatic dermatitis with ameliorated epidermal thickness, erythema, scaling and infiltration (Fig. 1h–k). Moreover, the I-A/I-E expression in the skin was also decreased after anti-MHC-II treatment (Fig. 1l), accompanied by a predominant T_H_17 cell percentage reduction in the IMQ-treated skin (Fig. 1m), indicating that MHC-II may facilitate the psoriasis process.

### Genomic deletion of H2-Ab1 in the epidermis alleviates psoriasis-like inflammatory responses

**Fig. 2:**
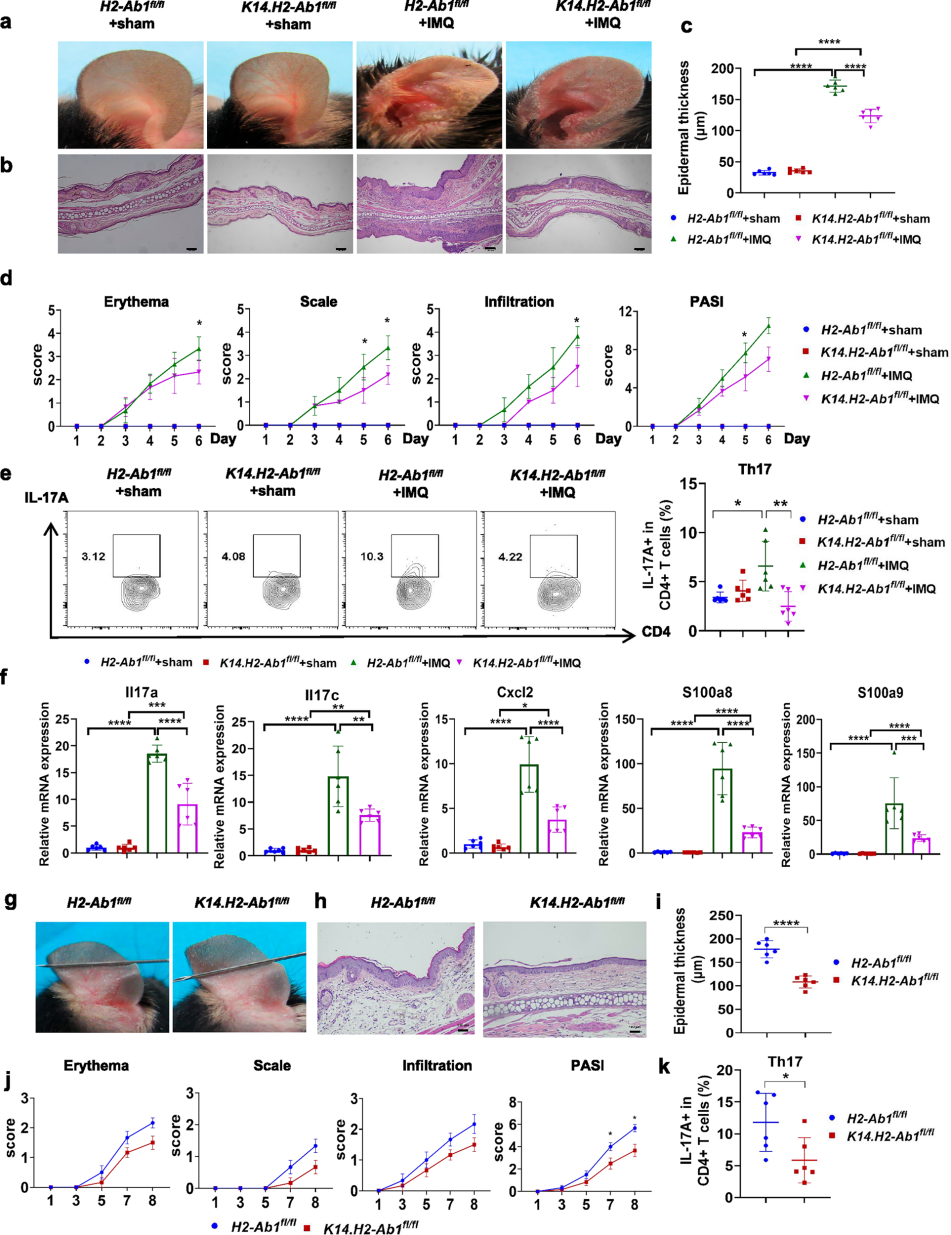
H2-Ab1 deficiency in epidermis alleviates IMQ-induced dermatitis. **a**–**d** Phenotypic presentation (**a**) and H&E staining (**b**) as well as statistical analysis of the epidermal thickness (**c**) and PASI scores (**d**) of the ear skin in *H2-Ab1*^*fl/fl*^ and *K14.H2-Ab1*^*fl/fl*^ mice treated with sham or IMQ. Scale bars, 100 μm. **e** Representative flow cytometric plots and quantification of skin T_H_17 cell percentage gated on the CD4^+^ T cells as indicated. **f** The mRNA expression of psoriasis-related factors in the skin as indicated. **g**–**j** Phenotypic presentation (**g**) and H&E staining (**h**) as well as statistical analysis of epidermal thickness (**i**) and PASI scores (**j**) of the ear skin in *H2-Ab1*^*fl/fl*^ and *K14.H2-Ab1*^*fl/fl*^ mice treated with IL-23. Scale bars, 100 μm. **k** Quantification of skin T_H_17 cell percentage gated on the CD4^+^ T cells as indicated. *n* = 6. Data are shown as the mean ± s.d. *P* values were determined using one-way ANOVA with Tukey’s multiple comparisons test or two-tailed Student’s *t*-test. *P < 0.05, **P < 0.01, ***P < 0.001, ****P < 0.0001.

Next, we constructed the epidermis-specific H2-Ab1 KO mice by crossing the *H2-Ab1*^*fl/fl*^ mice with the K14-cre mice (Supplementary Fig. 3a). The expression of H2-Ab1 was successfully deleted in the epidermis (Supplementary Fig. 3b–d). As expected, the H2-Ab1 deficiency in the epidermis significantly attenuated IMQ-induced psoriasis-like inflammatory responses (Fig. 2a). The psoriatic severity regarding redness, scaling and infiltration in skin lesions, as well as epidermal thickness, was markedly reduced in *K14.H2-Ab1*^*fl/fl*^ mice (Fig. 2b-d). The accumulation of T_H_17 cells as well as psoriasis-related molecules including Il17a, Il17c, Cxcl2, S100a8 and S100a9, was inhibited in the *K14.H2-Ab1*^*fl/fl*^ mice (Fig. 2e, f). In addition, the spleen index was also reduced in *K14.H2-Ab1*^*fl/fl*^ mice (Supplementary Fig. 3e). Meanwhile, H2-Ab1 deficiency in the epidermis did not affect skin thickness, inflammatory cell accumulation or the expression of psoriasis-related molecules under normal conditions (Fig. 2a–f).

We also performed the IL-23-induced psoriasis-like mouse model using *H2-Ab1*^*fl/fl*^ and *K14.H2-Ab1*^*fl/fl*^ mice. Consistent with the above results, the psoriatic phenotype was relieved in *K14.H2-Ab1*^*fl/fl*^ mice, compared with *H2-Ab1*^*fl/fl*^ mice (Fig. 2g–j). T_H_17 cell infiltration in skin lesions was also attenuated in the H2-Ab1 KO mice under IL-23 treatment (Fig. 2k). These results indicate that the epidermal expression of MHC-II plays essential functions in the psoriasis process.

### MHC-II expressed in KCs triggers antigen-dependent naive CD4^+^ T cell activation and proliferation

MHC-II expression could be enhanced in KCs under the stimulation of IFN-γ (Supplementary Fig. 4a-c), and IMQ treatment increased IFN-γ levels in skin tissue (Supplementary Fig. 4d). However, whether KCs functioned as APCs remained undefined. KCs alone were unable to activate naive CD4^+^ T cells isolated from the spleens of OT-II mice (Supplementary Fig. 4e). Following pretreatment with IFN-γ, KCs loaded with OVA resulted in the initiation and amplification of CD4^+^ T cell activation (Supplementary Fig. 4f). Moreover, KCs have features to enhance the proliferation of CD4^+^ T cells in an IFN-γ- and OVA-dependent manner (Supplementary Fig. 4g). However, the co-culture of KCs and naive CD4^+^ T cells did not affect T_H_17 cell differentiation (Supplementary Fig. 4h), even under T_H_17-skewing conditions (Supplementary Fig. 4i).

Next, primary KCs isolated respectively from the newborn *H2-Ab1*^*fl/fl*^ mice and *K14.H2-Ab1*^*fl/fl*^ mice were co-cultured with naive CD4^+^ T cells under IFN-γ and OVA pretreatment. As expected, KC-specific H2-Ab1 deficiency significantly restrained T cell activation and clonal expansion (Supplementary Fig. 4j,k), suggesting that the MHC-II molecule is required for the antigen presentation function of KCs.

### The KO of H2-Ab1 expression in the epidermis reshapes the cellular and functional landscape of IMQ-induced psoriatic dermatitis

**Fig. 3:**
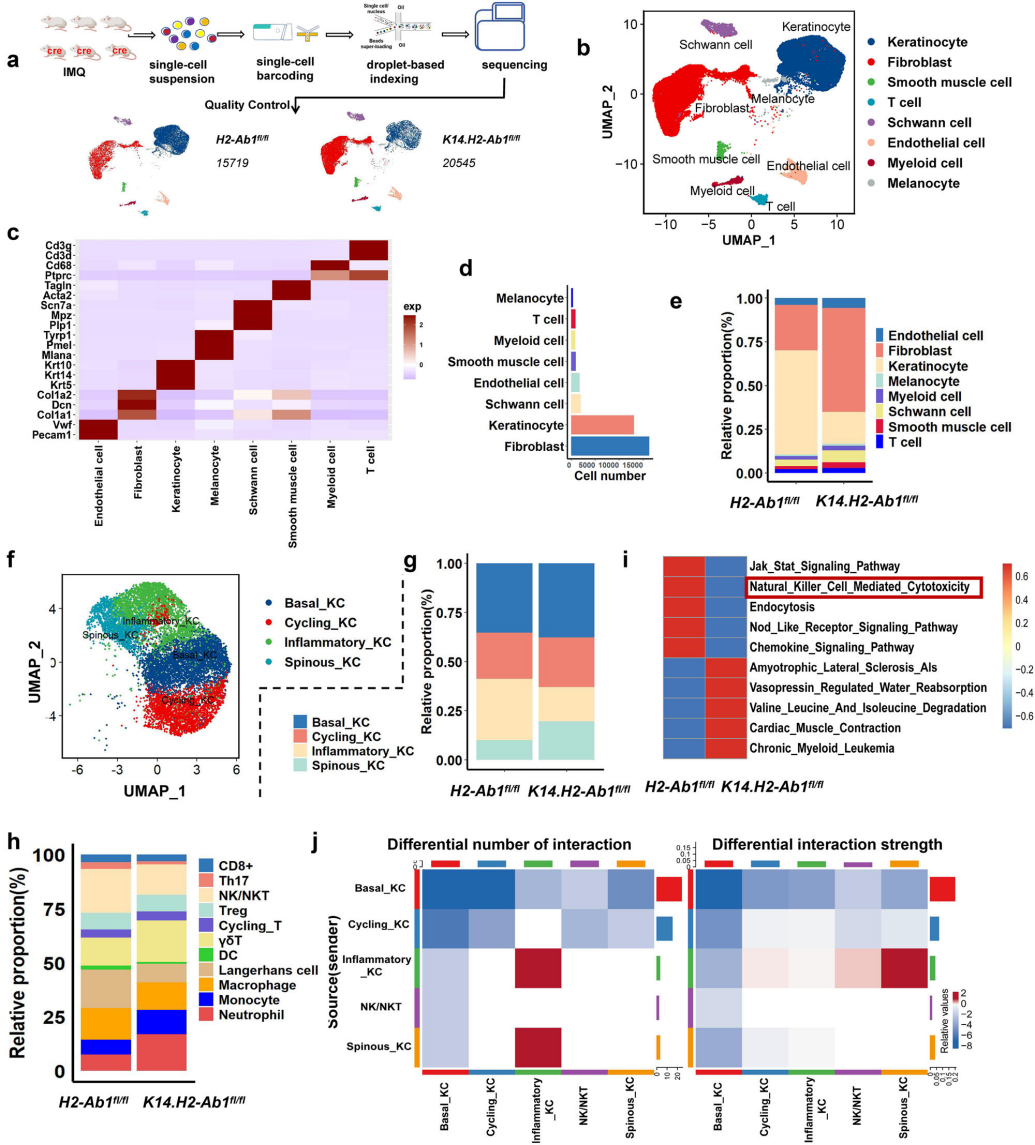
The cellular landscape of the IMQ-induced skin from the *H2-Ab1*^*fl/fl*^ and *K14.H2-Ab1*^*fl/fl*^ mice. **a** Workflow of scRNA-seq for ear skin samples from the *H2-Ab1*^*fl/fl*^ and *K14.H2-Ab1*^*fl/fl*^ mice treated with IMQ (*n* = 3). **b** Representative UMAP plot of major cell populations in the ear skin of IMQ-treated mice. **c** Feature plots of expression distribution for cluster-specific marker genes in the identified cell types revealed by the heatmap. **d** Cell number of the major cell subsets shown by the bar plot. **e** The relative frequency of the major cell types in the *H2-Ab1*^*fl/fl*^ and *K14.H2-Ab1*^*fl/fl*^ mice. **f** UMAP visualization of cell subpopulations of KCs. **g** The proportions of subpopulations of KCs in the *H2-Ab1* and *K14.H2-Ab1*^*fl/fl*^ mice. **h** The proportions of immune cell subpopulations in the *H2-Ab1*^*fl/fl*^ and *K14.H2-Ab1*^*fl/fl*^ mice. **i** Heatmap of enriched KEGG pathways across differentiated genes of NK/NKT cells in the *H2-Ab1*^*fl/fl*^ and *K14.H2-Ab1*^*fl/fl*^ mice using GSVA. **j** Heatmap showing the differential interaction number or strength between KC subsets and NK/NKT cells. The color on the heatmap indicates whether *H2-Ab1*^*fl/fl*^ or *K14.H2-Ab1*^*fl/fl*^ mice predominate in terms of the number or strength of interactions for each cell type pair (blue: *H2-Ab1*^*fl/fl*^; red: *K14.H2-Ab1*^*fl/fl*^).

Then, we performed scRNA-seq to analyze the cell landscapes and transcriptional alterations in *K14.H2-Ab1*^*fl/fl*^ mice with IMQ topical application (Fig. 3a). Many pivotal signaling pathways including the epidermal cell differentiation and IL-17 signaling pathway, were significantly enriched based on differentially expressed genes (DEGs) according to the scRNA-seq results (Supplementary Fig. 5a, b). Through Uniform Manifold Approximation and Projection (UMAP) plotting analysis, we identified eight cell clusters (Fig. 3b) based on the unique marker expressions (Fig. 3c). As previously reported, KCs and fibroblasts occupied the majority of the skin (Fig. 3d). The percentage of KCs was reduced in the *K14.H2-Ab1*^*fl/fl*^ mice (Fig. 3e). Further subclustering of KCs identified four subpopulations according to differently expressed feature genes (Fig. 3f and Supplementary Fig. 6a), with the decreased proportion of inflammatory KC in the *K14.H2-Ab1*^*fl/fl*^ mice (Fig. 3g). Moreover, KCs demonstrated a dynamic pseudo-temporal ordering from basal KC and cycling KC to inflammatory KC or spinous KC according to the transcriptional trajectory (Supplementary Fig. 6b–d). Key differentiation-related genes drove this progression (Supplementary Fig. 6e, f), while branch-dependent genes (for example, Krt17, Krt6a and Krt6b) influenced cell fate decisions (Supplementary Fig. 6g). Moreover, different trajectory structures were detected in KCs of *H2-Ab1*^*fl/fl*^ and *K14.H2-Ab1*^*fl/fl*^ mice (Supplementary Fig. 6h). MHC-II-deficient KCs inhibited abnormal epidermal differentiation owing to decreased retention of basal KC and cycling KC in the late stages of KC development for *K14.H2-Ab1*^*fl/fl*^ mice (Supplementary Fig. 6i).

In myeloid cells, we identified five distinct clusters composed of DCs, Langerhans cells, macrophages, monocytes and neutrophils (Supplementary Fig. 7a), and each cell subpopulation was defined by characteristic markers (Supplementary Fig. 7b). T cells were further separated into γδT, T_H_17, cycling T, T_reg_, NK/NKT and CD8^+^ T cells (Supplementary Fig. 7c,d). Among these immune cells, proportions of DCs, Langerhans cells, T_H_17 cells and NK/NKT cells were predominantly reduced in the *K14.H2-Ab1*^*fl/fl*^ mice (Fig. 3h). The roles of DCs, Langerhans cells and T_H_17 cells in the pathogenesis of psoriasis have been well known, while the function of NK and NKT cells in psoriasis remains unclear.

Based on the gene set variation analysis (GSVA) results, functional states especially cytotoxic characteristics of NK/NKT cells were significantly inhibited after specific KO of H2-Ab1 in KCs (Fig. 3i). CellChat analysis showed a reduction in both the number and strength of cell interactions in the *K14.H2-Ab1*^*fl/fl*^ mice (Supplementary Fig. 8a). KCs mainly established mutual interaction with immune cells under the IMQ-induced psoriasis-like condition (Supplementary Fig. 8b). In particular, the number and strength of interactions between KC–NK/NKT cell pairs, especially basal-KC–NK/NKT pairs, were significantly reduced in *K14.H2-Ab1*^*fl/fl*^ mice (Fig. 3j).

### Depletion of NK/NKT cells by an inhibitor attenuates the IMQ-induced psoriasis-like phenotype

**Fig. 4:**
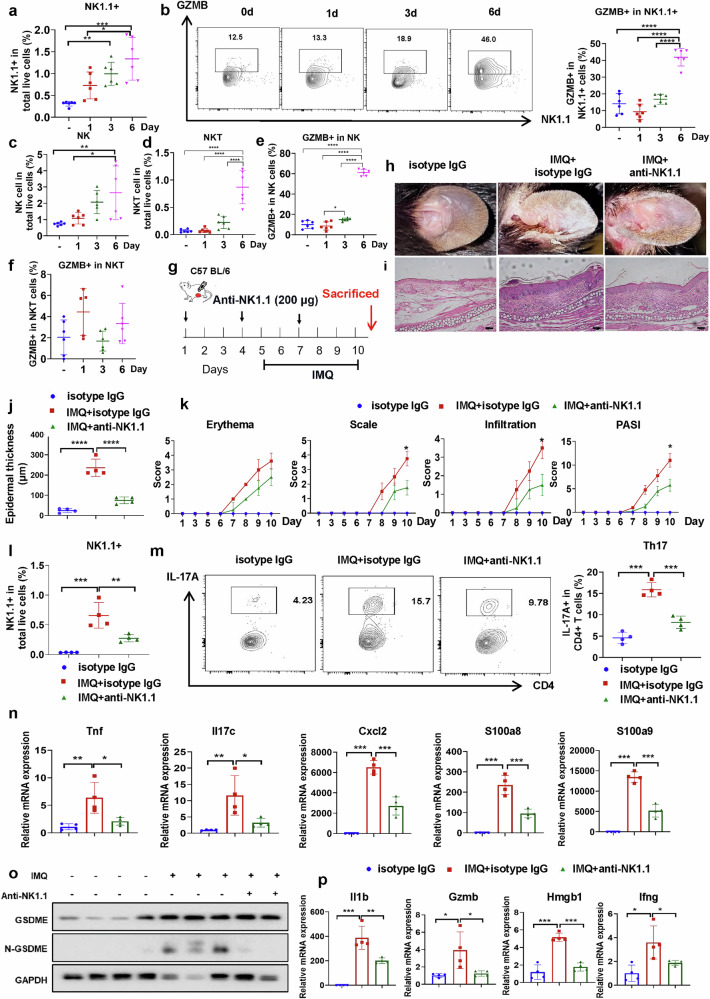
Depletion of NK1.1 attenuates IMQ-induced psoriatic inflammation. **a**, **b** Flow cytometric analysis of the percentage of NK1.1^+^ cells gated on the total live cells (**a**) and the GZMB release ratio gated on the NK1.1^+^ cells (**b**) as indicated. *n* = 6. **c**–**f** Flow cytometric analysis of the percentage of NK (**c**) or NKT cells (**d**) gated on the total live cells and the GZMB release ratio gated on the NK (**e**) or NKT cells (**f**) as indicated. *n* = 6. **g** Schematic diagram of anti-NK1.1 intraperitoneal administration (200 μg) in mice with IMQ topical application. *n* = 4. **h**–**k** Phenotypic presentation (**h**) and H&E staining (**i**) as well as statistical analysis of the epidermal thickness (**j**) and PASI scores (**k**) of the ear skin in the control or IMQ-treated mice with isotype IgG or anti-NK1.1 application. Scale bars, 100 μm. **l**, **m** Representative flow cytometric analysis of the NK1.1^+^ cell percentage gated on total live cells (**l**) or T_H_17 cell percentage gated on the CD4^+^ T cells (**m**) as indicated. **n** The mRNA expression of psoriasis-related factors in the skin as indicated. **o** Immunoblot for GSDME and its cleavage form in skin lysates. **p** The mRNA expression of pyroptosis-related factors in the skin as indicated. Data are shown as the mean ± s.d. *P* values were determined using one-way ANOVA with Tukey’s multiple comparisons test or two-tailed Student’s *t*-test. *P < 0.05, **P < 0.01, ***P < 0.001, ****P < 0.0001.

As KC-specific H2-Ab1 expression impaired proportions and functions of NK/NKT revealed by scRNA-seq results, we investigated the role of NK/NKT (expressing NK1.1 markers in flow cytometry) in the IMQ-induced mouse model and discovered the NK1.1^+^ cell accumulation and the GZMB released from NK1.1^+^ cells had been gradually raised during consecutive IMQ topical application (Fig. 4a, b). Both NK and NKT cell percentages were elevated in the IMQ-treated skin tissues (Fig. 4c, d) while only GZMB from NK cells had been remarkably increased with IMQ applications for 6 days (Fig. 4e, f), indicating that NK1.1^+^ cells, especially NK cells, might play a cytotoxic role in controlling their capacity to release GZMB, thereby exacerbating the development of psoriasis.

Then, we administered anti-NK1.1 antibodies to IMQ-induced psoriasis-like mice following a predefined time schedule (Fig. 4g). Depletion of NK1.1^+^ cells significantly abrogated the progression of psoriasis-like pathology induced by IMQ (Fig. 4h), as indicated by the reduced Psoriasis Area and Severity Index (PASI) score and decreased epidermal thickness (Fig. 4i–k). Moreover, the depletion of NK1.1^+^ cells by anti-NK1.1 antibody (Fig. 4l) significantly reduced T_H_17 cell responses (Fig. 4m) and mRNA expression of psoriasis-related inflammatory molecules, such as IL-17c, S100a8 and S100a9 (Fig. 4n).

A recent report showed that GZMB could cleave gasdermin E (Gsdme) and form nonselective pores on the cell surface, ultimately leading to pyroptosis^25^. The cleavage of GSDME was significantly elevated in IMQ-induced psoriasiform skin (Fig. 4o), whereas anti-NK1.1 antibody suppressed GSDME-related pyroptosis (Fig. 4o) as well as the mRNA levels of pyroptosis-related molecules, including Il1b, Ifng, Gzmb and Hmgb1 (Fig. 4p), indicating that NK/NKT cell-mediated pyroptosis exerts a vital role in psoriasis.

### Deletion of MHC-II in KCs reduces NK cell recruitment through the regulation of CXCL10 via the ERK1/2–CREB axis

**Fig. 5:**
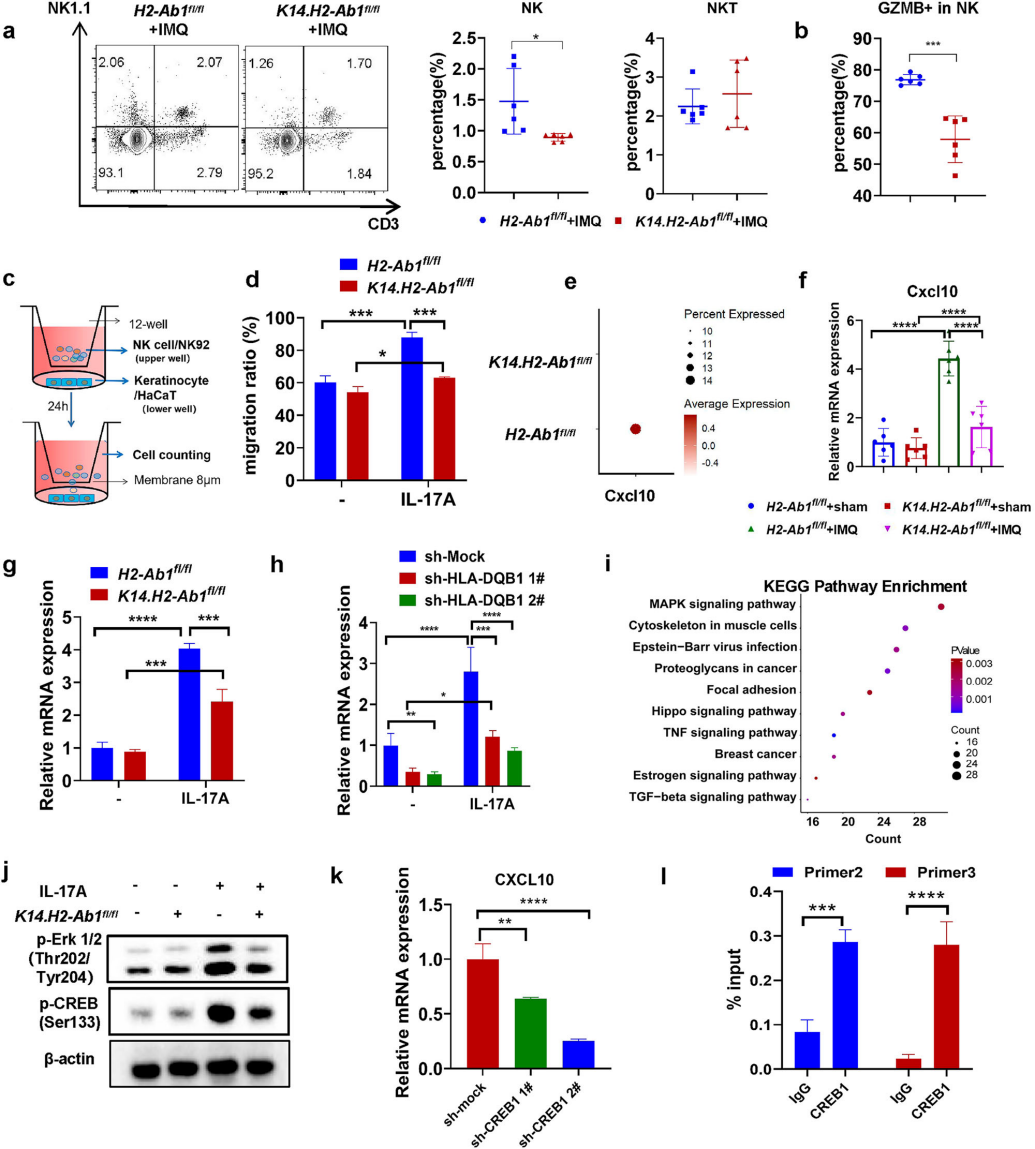
Inhibition of H2-Ab1 in the KCs suppresses NK cell migration through the CREB–CXCL10 axis. **a** Representative flow cytometric plots and quantification of skin NK (top-left quadrants) or NKT cell (top-right quadrants) percentage. *n* = 6. **b** Flow cytometric quantification of the GZMB abundance in NK cells. *n* = 6. **c** Schematic diagram of the KC–NK cell co-culture systems. **d** The migration ratio of NK cells co-cultured with KCs from *H2-Ab1*^*fl/fl*^ and *K14.H2-Ab1*^*fl/fl*^ mice stimulated with or without IL-17A for 24 h. **e** Cxcl10 expression in the KC cluster based on the scRNA-seq analysis of IMQ-treated skin. **f** Cxcl10 mRNA level in the epidermis of *H2-Ab1*^*fl/fl*^ and *K14.H2-Ab1*^*fl/fl*^ mice treated with or without IMQ for 6 days. *n* = 6. **g** Cxcl10 mRNA expression in KCs isolated from the newborn *H2-Ab1*^*fl/fl*^ mice and *K14.H2-Ab1*^*fl/fl*^ mice with or without IL-17A stimulation for 24 h. **h** CXCL10 mRNA level in HaCaTs infected with vector or HLA-DQB1 lentivirus. **i** The KEGG enrichment analysis of KCs between *H2-Ab1*^*fl/fl*^ and *K14.H2-Ab1*^*fl/fl*^ mice based on DEGs from scRNA-seq results. **j** Cell lysates were extracted from KCs isolated from the newborn *H2-Ab1*^*fl/fl*^ mice and *K14.H2-Ab1*^*fl/fl*^ mice with or without IL-17A stimulation for 30 min; immunoblotting for p-Erk 1/2 and p-CREB levels in KCs was then performed. **k** CXCL10 mRNA level in HaCaTs infected with vector or CREB1 lentivirus. **l** ChIP–quantitative PCR (qPCR) assays for evaluating CREB1 binding to the CXCL10 promoter sites in HaCaTs. Data in **d**, **g**, **h**, **j** and **k** are representative of three independent experiments. Data are shown as the mean ± s.d. *P* values were determined using one-way ANOVA with Tukey’s multiple comparisons test or two-tailed Student’s *t*-test. *P < 0.05, **P < 0.01, ***P < 0.001, ****P < 0.0001.

Next, we examined the percentage of NK (CD3⁻NK1.1⁺) or NKT(CD3⁺NK1.1⁺) cells in *K14.H2-Ab1*^*fl/fl*^ mice under IMQ-induced psoriasis inflammation. The percentages of NK cells (rather than NKT cells) were decreased in these mice compared with the control mice (Fig. 5a). Moreover, GZMB release from NK cells was also reduced in epidermal-specific H2-Ab1-deficient mice under IMQ treatment (Fig. 5b).

To confirm MHC-II’s role in KC-mediated NK cell recruitment, we established a KC–NK cell co-culture system in vitro (Fig. 5c). We generated HLA-DQB1 (human MHC-II gene, corresponding to H2-Ab1) knockdown HaCaTs (Supplementary Fig. 9a, b) and then co-cultured these HaCaTs with NK92. The result showed the knockdown of HLA-DQB1 in HaCaTs significantly inhibited the NK92 migration under the treatment of IL-17A (Supplementary Fig. 9c). We also isolated KCs from the *H2-Ab1*^*fl/fl*^ and *K14.H2-Ab1*^*fl/fl*^ mice, which were co-cultured with primary NK cells, sorted from the spleen (purity >95%; Supplementary Fig. 9d). As shown in Fig. 5d, deletion of H2-Ab1 in KCs reduced the migration of NK cells.

To determine whether KC-mediated NK cell migration was linked to impaired chemokine signaling, we analyzed scRNA-seq data and found a significant reduction in Cxcl10 mRNA levels in the KC cluster of *K14.H2-Ab1*^*fl/fl*^ mice (Fig. 5e). CXCL10 is a key chemokine implicated in both NK cell chemotaxis and psoriasis pathogenesis^26,27^, and its transcriptional expression was increased in the KC cluster of psoriatic skin lesions (Supplementary Fig. 9e, f) and decreased in the epidermis of *K14.H2-Ab1*^*fl/fl*^ mice after IMQ treatment (Fig. 5f). Meanwhile, under IL-17A stimulation, the mRNA expression of CXCL10 was downregulated in HaCaTs with HLA-DQB1 knockdown or primary KCs isolated from *K14.H2-Ab1*^*fl/fl*^ mice (Fig. 5g, h). Therefore, MHC-II deficiency in epidermal KCs might impair IMQ-induced NK cell recruitment by downregulating CXCL10 expression.

Subsequently, we investigated the possible mechanism of how MHC-II upregulated CXCL10 expression in KCs. Enriched KEGG pathway and GSVA analysis indicated that the MAPK signaling pathway was differentially regulated in KCs after H2-Ab1 KO (Fig. 5i and Supplementary Fig. 9g). Depletion of H2-Ab1 in the epidermis dramatically attenuated IL-17A induced activation of ERK1/2 and cyclic AMP response-element binding protein (CREB), but not that of p38 or JNK1/2 (Fig. 5j and Supplementary Fig. 9h). Knocking down expression of CREB1 (Supplementary Fig. 9i) inhibited the CXCL10 transcriptional expression (Fig. 5k). Meanwhile, ChIP results indicated that CREB1 could directly bind to the promoter of CXCL10 (Fig. 5l and Supplementary Fig. 9j, k). Taken together, MHC-II in KCs could transcriptionally regulate CXCL10 expression mediated by CREB1, consequently recruiting NK cells in psoriasis skin lesions.

### GSDME-mediated pyroptosis facilitates the pathogenesis of psoriasis

**Fig. 6:**
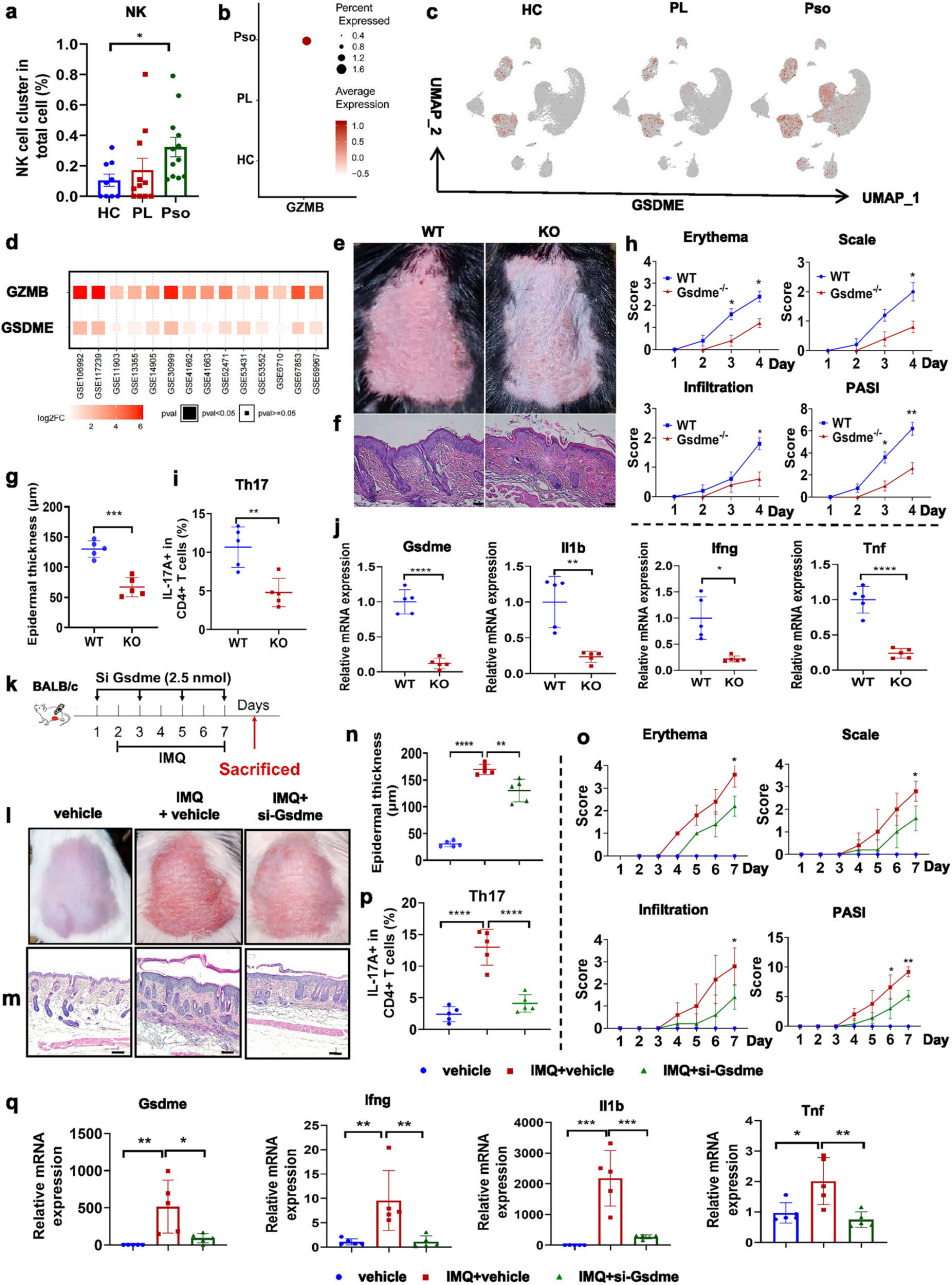
Gsdme deficiency prevents the facilitation of IMQ-induced psoriasis-like dermatitis. **a**–**c** scRNA-seq analysis of NK cell percentage (**a**), GZMB abundance (**b**) and the GSDME expression projected on the UMAP plot (**c**) under different skin conditions based on datasets GSE162183 and GSE173706 (healthy skin (HC), paralesional skin (PL) and psoriatic skin (Pso)). **d** Bioinformatic analysis of GZMB and GSDME expression in psoriatic lesions compared with healthy skin using psoriasis GEO data. **e**–**h** Phenotypic presentation (**e**) and H&E staining (**f**) as well as statistical analysis of the epidermal thickness (**g**) and PASI scores (**h**) of back skin in *Gsdme*^−/−^ and WT mice with IMQ treatment. Scale bars, 100 μm. **i** Flow cytometric quantification of the T_H_17 percentage gated on the CD4^+^ T cells as indicated. **j** The mRNA expression of pyroptosis-related factors in the skin of *Gsdme*^−/−^ and WT mice with IMQ treatment. **k** Schematic diagram of si-Gsdme topical application (2.5 nmol) every other day in the mice with IMQ treatment. **l**–**o** Phenotypic presentation (**l**) and H&E staining (**m**) as well as statistical analysis of the epidermal thickness (**n**) and PASI scores (**o**) of back skin in the control or IMQ-treated mice with or without si-Gsdme topical application. Scale bars, 100 μm. **p** Flow cytometric quantification of skin T_H_17 cells gated on the CD4^+^ T cells as indicated. **q** The mRNA expression of pyroptosis-related factors in the back skin as indicated. *n* = 5. Data are shown as the mean ± s.d. *P* values were determined using one-way ANOVA with Tukey’s multiple comparisons test or two-tailed Student’s *t*-test. *P < 0.05, **P < 0.01, ***P < 0.001, ****P < 0.0001.

Furthermore, we confirmed the increased NK cell percentage (Fig. 6a) and GZMB abundance in psoriasis skin lesions (Fig. 6b), consistent with the public scRNA-seq data. The GSDME level was increased in psoriatic skin cell populations (Fig. 6c). The transcriptional expression of GZMB and GSDME was also upregulated in psoriatic skin, as revealed by analysis of the psoriasis GEO database (Fig. 6d), supporting the important role of GZMB-mediated GSDME activation during psoriasis pathogenesis.

Then, we applied the pyroptosis inhibitor Z-DEVD-FMK to the IMQ-induced psoriasis-like mouse model (Supplementary Fig. 10a), which blocked the cleavage of GSDME and indirectly inhibited pyroptosis. Interestingly, the application of Z-DEVD-FMK significantly alleviated the psoriatic phenotype (Supplementary Fig. 10b), including epidermal thickness and PASI scores (Supplementary Fig. 10c–e). Cleavage of GSDME in the skin was inhibited after the Z-DEVD-FMK treatment (Supplementary Fig. 10f), which also abrogated IMQ-induced psoriasis-like inflammatory responses characterized by decreased T_H_17 cell infiltration (Supplementary Fig. 10g) as well as reduced mRNA levels of pyroptosis-related molecules (Supplementary Fig. 10h).

To further investigate the effects of GSDME-mediated pyroptosis on psoriasis, we constructed the IMQ-induced psoriasis-like mouse model on the Gsdme^−/−^ (KO) mice. The expression of Gsdme was significantly blocked in the *Gsdme* KO mice, identified by rtPCR and WB (Supplementary Fig. 11a, b). *Gsdme*^*−/−*^ mice presented significantly alleviated psoriatic skin lesions compared with WT mice (Fig. 6e). The epidermal thickness (Fig. 6f, g) as well as the PASI scores (Fig. 6h) were remarkably reduced in *Gsdme*^−/−^ mice. T_H_17 cell infiltration (Fig. 6i) as well as pyroptosis- and psoriasis-related molecules (Fig. 6j and Supplementary Fig. 11c, d) were also suppressed in skin lesions of Gsdme KO mice. The KO of Gsdme proved the similar alleviative effects on impeding the psoriatic pathological progression in the IL-23-induced psoriasis-like mouse model (Supplementary Fig. 11e–j).

Then, we validated the suppression effect on Gsdme expression by si-Gsdme (Supplementary Fig. 11k, l) and topically applied the siRNA targeting Gsdme on the IMQ-treated mice for inhibiting the Gsdme expression in the skin (Fig. 6k). The GSDME expression suppression blocked IMQ-mediated psoriasis-like dermatitis (Fig. 6l). The epidermal thickness and the psoriatic phenotype, including redness, scaling and thick infiltration, were significantly reduced in the si-Gsdme-treated mice (Fig. 6m–o). The accumulating T_H_17 cells in the IMQ-induced skin were suppressed after si-Gsdme topical application (Fig. 6p). Inhibition of GSDME expression in the skin by siRNA also reduced mRNA levels of pyroptosis-related molecules, including Il1b and Tnf (Fig. 6q), as well as psoriasis-related factors, such as Cxcl2 and S100a8 (Supplementary Fig. 11m). The above results confirmed the pivotal role of GSDME-mediated pyroptosis in driving the inflammatory changes associated with psoriasis.

### NK cells predominantly target KCs to induce the GSDME-mediated pyroptosis through GZMB

**Fig. 7:**
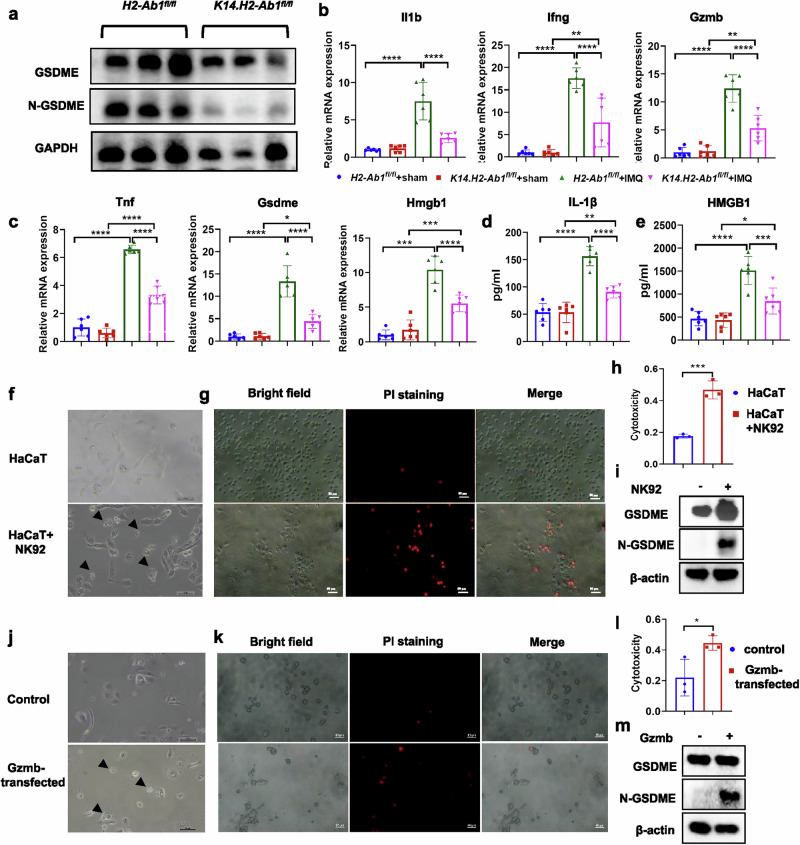
MHC-II in KCs enhances NK cell-targeted pyroptosis in IMQ-induced psoriasis-like epidermis. **a** Immunoblot for GSDME and its cleavage form in skin lysates of *H2-Ab1*^*fl/fl*^ and *K14.H2-Ab1*^*fl/fl*^ mice treated with IMQ. *n* = 3. **b**, **c** The mRNA expression of pyroptosis-related factors in the skin of *H2-Ab1*^*fl/fl*^ and *K14.H2-Ab1*^*fl/fl*^ mice treated with or without IMQ. *n* = 6. **d**, **e** The secretion of IL-1β (**d**) and HMGB1 (**e**) in the skin of *H2-Ab1*^*fl/fl*^ and *K14.H2-Ab1*^*fl/fl*^ mice treated with or without IMQ measured by ELISA. *n* = 6. **f** Phase-contrast images of cell morphology for HaCaTs co-cultured with or without NK92. **g** HaCaTs co-cultured with or without NK92 were stained with PI and visualized under bright field or the fluorescence microscopy. **h** The cell death level of HaCaTs co-cultured with or without NK92 was measured by LDH. **i** Cell lysates were extracted from HaCaTs co-cultured with or without NK92. Immunoblot for the GSDME and its cleavage form was then performed. **j** Phase-contrast images of cell morphology for primary KCs transfected with or without GZMB. **k** Primary KCs transfected with or without GZMB were stained with PI and visualized under bright-field or fluorescence microscopy. **l** The cell death level of primary KCs transfected with or without GZMB was measured by LDH. **m** Cell lysates were extracted from primary KCs transfected with or without GZMB. Immunoblot for the GSDME and its cleavage form was then performed. Data in **f**–**m** are representative of at least three independent experiments. Data are shown as the mean ± s.d. *P* values were determined using one-way ANOVA with Tukey’s multiple comparisons test or two-tailed Student’s *t*-test. *P < 0.05, **P < 0.01, ***P < 0.001, ****P < 0.0001.

Given that GSDME is a key molecule to initiate the pyroptosis procedure, we tested the effect of epidermal expression of MHC-II on the activation of GSDME. As expected, the cleavage of GSDME was significantly blocked in the skin of *K14.H2-Ab1*^*fl/fl*^ mice, compared with the control mice (Fig. 7a). Moreover, under IMQ-induced psoriatic conditions, *K14.H2-Ab1*^*fl/fl*^ mice exhibited significantly reduced mRNA expression of pyroptosis-related genes and lower secretion levels of IL-1β and HMGB1 in the skin (Fig. 7b–e), consistent with the scRNA-seq results that Ucell scores for pyroptosis-related gene signatures were reduced after specific H2-Ab1 KO in epidermis (Supplementary Fig. 12a).

However, it is still necessary to determine which cell type was the major target of NK cells in MHC-II-mediated GSDME-related pyroptosis alterations. GSDME was mainly expressed in fibroblasts and KCs (especially basal KC) (Supplementary Fig. 12b, c). Based on the mouse scRNA-seq results, pyroptosis level scores were significantly higher in KCs than in fibroblasts (Supplementary Fig. 12d). Furthermore, in the CellChat analysis, NK/NKT predominantly established cell–cell communication with KCs rather than fibroblasts (Supplementary Fig. 12e). The number and strength of interactions were reduced, particularly in basal KCs of *K14.H2-Ab1*^*fl/fl*^ mice, when NK/NKT cells acted as the major signal inputs (Supplementary Fig. 12f).

To verify the spatial orientation and connection between NK cells and KCs, we conducted the spatial transcriptomic sequencing using IMQ-induced ear skin samples from *H2-Ab1*^*fl/fl*^ and *K14.H2-Ab1*^*fl/fl*^ mice. Major cell populations were identified in the spatial context. Generally, the KC cluster was predicted to be preferentially in the epidermal layers of the skin tissues, while the fibroblast cluster was predicted to predominantly occupy the dermal region of the skin tissues (Supplementary Fig. 13a). NK cells were located in the epidermal compartment around the KC cluster but were detected at low levels in the skin tissues of *K14.H2-Ab1*^*fl/fl*^ mice (Supplementary Fig. 13b). Nkg7, Gzmb and Gsdme expression was mapped mostly within epidermal regions (Supplementary Fig. 13c), with a co-expression or local distribution in the lower layer of the epidermis or the vicinity of basal KCs (Supplementary Fig. 13d). Intercellular communications between KCs and NK cells were more predominant in *H2-Ab1*^*fl/fl*^ mice compared with *K14.H2-Ab1*^*fl/fl*^ mice (Supplementary Fig. 13e).

Moreover, HaCaTs co-cultured with NK92 promoted cell death and pyroptotic pores (Fig. 7f), measured as increased PI uptake (Fig. 7g) and lactate dehydrogenase (LDH) release (Fig. 7h). The expression of N-GSDME was also enhanced in the KC/NK cell co-culture condition (Fig. 7i). KCs transfected with GZMB exhibited pyroptosis-related cell death morphology, accompanied by increased PI uptake, LDH release and elevated N-GSDME expression (Fig. 7j–m).

### MHC-II in the epidermis drives the skin trafficking of NK cells by CXCL signaling and induces subsequent GSDME-elicited pyroptosis in psoriatic skin lesions

**Fig. 8:**
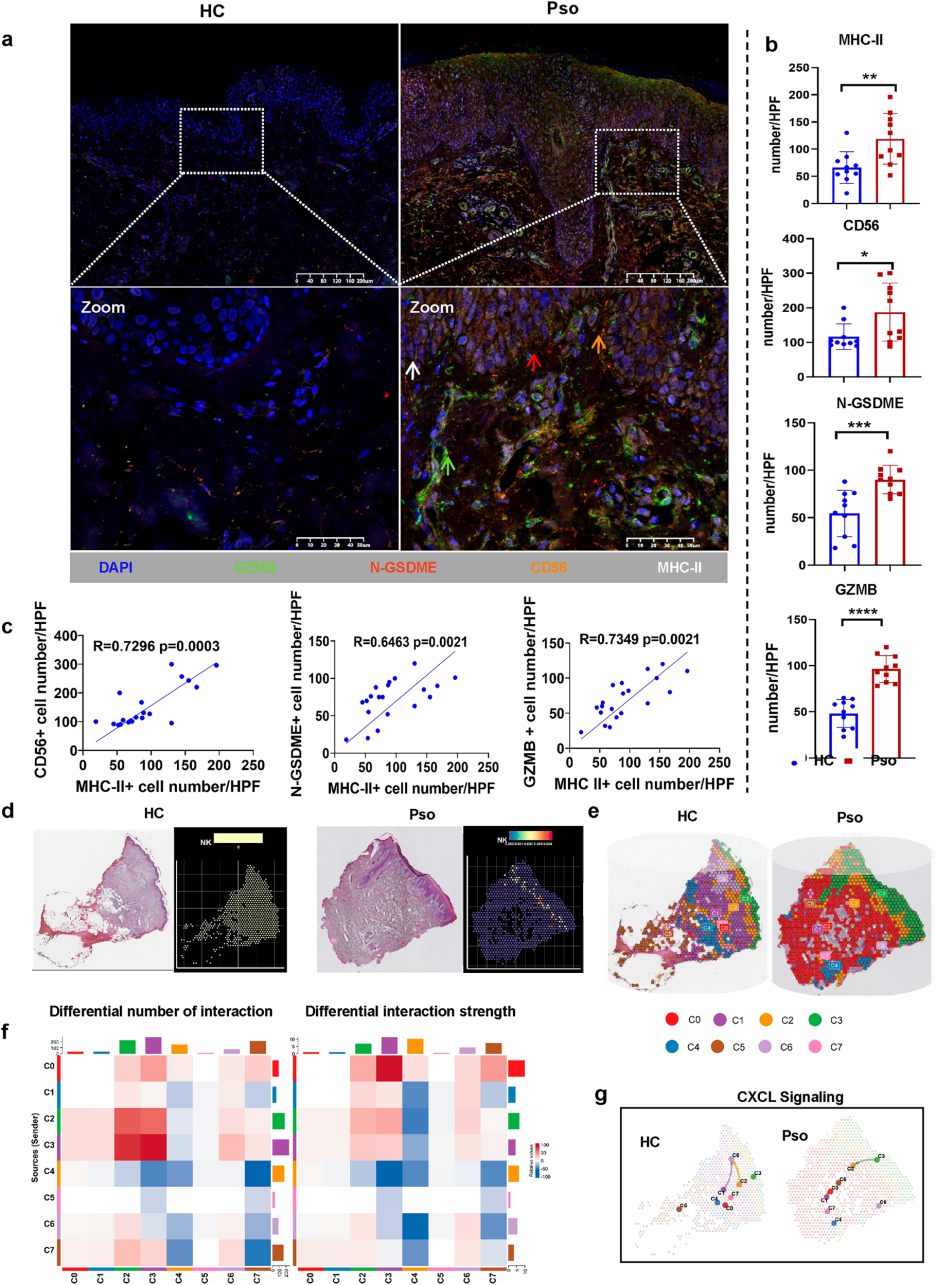
Epidermal MHC-II enhances NK cell migration, GZMB release and subsequent GSDME-mediated pyroptosis in psoriatic epidermis. **a**, **b** Representative multiple immunohistochemical staining images at 100× magnification (**a**) and the statistical analysis of expression levels of MHC-II in the epidermis and CD56, GZMB and N-GSDME in the skin of HC (*n* = 10) and psoriasis (Pso) (*n* = 10) (**b**). MHC-II^+^ cells are in white, CD56^+^ cells are in orange, GZMB^+^ cells are in green, and N-GSDME^+^ cells are in red. **c** The correlations of MHC-II^+^ cell number in epidermis and CD56^+^, N-GSDME^+^ and GZMB^+^ cell numbers in whole skin. **d** Representative H&E image and corresponding spatial distribution of NK cells in skin samples from HC and Pso. **e** Cell clustering analysis for cells from Pso and HC skin tissues. **f** Heatmaps showing the differential interaction number or strength among cell clusters. The color on the heatmap indicates which group predominates in the number or strength of interactions for each cell type pair (blue, HC; red, Pso). **g** Communication quantities of CXCL signaling pathway network among cell clusters in skin samples from HC and Pso. Panels **d**–**g** are based on the spatial transcriptomic data from GSE202011. Data are shown as the mean ± s.d. *P* values were determined using one-way ANOVA with Tukey’ s multiple comparisons test or two-tailed Student’s *t*-test. *P < 0.05, **P < 0.01, ***P < 0.001, ****P < 0.0001.

To further validate the relationships between MHC-II and NK cell-mediated pyroptosis in psoriasis, we examined the expression of MHC-II, CD56 (NK cell characteristic marker), GZMB and cleaved-GSDME in psoriasis and healthy skin samples by using multicolor immunohistochemistry (Fig. 8a and Supplementary Fig. 14a). As expected, the expression of MHC-II^+^ in the epidermis was elevated in psoriatic skin tissues, while the numbers of CD56^+^, GZMB^+^ and cleaved-GSDME^+^ cells in the skin were also markedly increased in individuals with psoriasis compared with HCs (Fig. 8b). Most importantly, we found significant positive correlations between the expression of MHC-II in the epidermis and the levels of CD56, GZMB and cleaved-GSDME in the psoriasis skin lesion (Fig. 8c). In the epidermal region, colocalization of CD56^+^GZMB^+^ NK cells with N-GSDME^+^ cells was observed, supporting the role of NK cells in inducing pyroptosis in KCs (Supplementary Fig. 14b).

The public spatial transcriptomic data for patients with psoriasis confirmed the increased expression of MHC-II in the epidermis (Supplementary Fig. 15). Cell type identification in the spatially resolved regions (Supplementary Figs. 16–18) revealed that NK cells were preferentially located near the basal layer of the epidermis and increased in psoriatic lesions (Fig. 8d). Furthermore, graph-based clustering of skin spots was classified into eight cell clusters (C0–C7) (Fig. 8e), of which C2 and C3 had enhanced mutual cell communications in patients with psoriasis (Fig. 8f). C3 was mainly located in the epidermal region, predominantly composed of KCs while NK cells belonged to C2. As our above data supported that KCs mediated the NK cell migration under the chemotactic role of CXCL10 in psoriasis, we further validated and extended our findings on how CXCL signaling coordinates cellular communication in a spatial context. Psoriatic lesions showed enhanced CXCL signaling (Supplementary Fig. 19), particularly from C3 to C2 (Fig. 8g). These results suggest that CXCL signaling may mediate the recruitment of C2 cluster-resident cells (including NK cells) to KC-rich regions, laying the foundation for subsequent KC–NK cell crosstalk.

## Discussion

MHC-II has been well documented to be responsible for presenting antigens to CD4^+^ T cells, which are crucial components of the adaptive immune system^28^. Previous studies have shown that KCs expressing MHC-II have antigen-presentation functions during IFN-γ stimulation^29^. Consistent with these results, our findings also indicated that KCs with genomic deletion of MHC-II failed to activate and expand T cells, thereby losing their antigen-presenting function. However, in recent years, accumulating evidence has shown that MHC-II exerts non-antigen-presenting functions in the pathogenesis of various diseases, including autoimmune disorders, infectious diseases and cancer^30–32^. For example, in the gastrointestinal tract, the MHC-II molecule coordinates the spatial segregation of the microbiota and epithelium in the gut that prevents inflammatory responses through colonization resistance against pathogens, which plays a crucial role in maintaining gut homeostasis and host–microbiota symbiosis^30,31^. MHC-II^+^ KCs exhibit enhanced functional activities, including antimicrobial peptide production, lymphocyte recruitment, antiviral responses, and AHR and IFN signaling^33^. In our study, MHC-II was highly expressed in psoriatic epidermal lesions and the deletion of H2-Ab1 in KCs significantly inhibited proliferation and abnormal differentiation of KCs as well as functional programs associated with inflammation, thus abrogating IMQ-mediated psoriasis-like inflammatory responses and reshaping the skin microenvironment.

Based on the scRNA-seq and in vivo results, H2-Ab1 expressed in the epidermis was revealed to influence the skin immune cell proportions, especially NK cells, not merely T_H_17 cells and DCs. NK cells are thought to build a bridge between innate and adaptive immunity. However, the role of NK cells in the pathogenesis of psoriasis remains controversial, partly due to the complexity of the receptors on these cells^34^. In our study, IMQ treatment could significantly recruit NK cell infiltration in psoriasis-like skin lesions, while genomic deletion of MHC-II in KCs blocked IMQ-induced NK cell infiltration through CXCL10, which has been demonstrated to be involved in NK cell chemotaxis and the pathogenesis of psoriasis^26,27^. Spatial transcriptomics identified that NK cells were located in the epidermal compartment or around the basal layer region. CXCL signaling participated in the intercellular interaction of the specific skin cell clusters within which NK cells and KCs existed. The above evidence validated a novel relationship between KCs and NK cells during the pathogenesis of psoriasis. Whether the interaction between KCs and NK cells is influenced by other cells, particularly T_H_17 cells, requires further investigation.

NK cells are widely acknowledged for their effector functions, including cytokine production and cell cytotoxicity^35,36^. The hallmark cytokine of NK cells is IFN-γ, which induces MHC-II expression in various cell types including DCs and KCs, potentially exacerbating MHC-II-mediated pathological progression in psoriasis^37^. NK cells can also target and eliminate virally infected or cancerous cells through their cytotoxic properties mediated by the perforin, granzymes and granulysin (GNLY)^38^. GZMB is an important molecule expressed primarily by NK and CTL cells^39^. Beyond its pro-apoptotic role, GZMB also cleaves matrix proteins, disrupts the epithelial barrier and increases vascular permeability, which facilitates the development of skin inflammation^40^. Consistent with previous reports of elevated GZMB in psoriatic lesions, our study confirmed that NK cell-derived GZMB is upregulated in psoriasis-like skin lesions during the late stage of IMQ treatment^41^, suggesting a potential role in amplifying the psoriasiform inflammatory response. Nevertheless, it remains to be investigated how GZMB shapes T_H_17 immunity and drives psoriatic pathogenesis and progression.

Recently, GZMB has been shown to have a novel role in cell death, inducing pyroptosis either by directly cleaving GSDME at the D270 residue or indirectly by activating caspase-3 to generate N-terminal fragments^25,42^. Pyroptosis is a type of programmed cell death linked to the activation of inflammatory and immune responses^43,44^. Accumulating evidence has revealed that pyroptosis exerts a critical role in skin inflammation. GSDMA has been identified as a key player in sensing and promoting pyroptosis in KCs during the recognition of virulence from microbial pathogens such as *Streptococcus pyogenes*^45^. GSDME in KCs contributes to the inflammatory progression of psoriasis by promoting the nuclear translocation of p65 and c-jun and production of psoriatic inflammatory mediators^46^, consistent with our findings that inhibiting GSDME cleavage or KO of Gsdme expression dramatically attenuated IMQ-mediated or IL-23-induced psoriasis-like dermatitis. The release of inflammatory mediators following pyroptosis, such as IL-1β and HMGB1, activates the IL-23–IL-17 signaling axis and establishes a positive feedback loop that perpetuates and amplifies the inflammatory state in psoriasis^47,48^. Therefore, the early DC/T cell-driven inflammatory microenvironment in psoriatic skin recruits and activates cutaneous NK cells^49–51^, exacerbating the KC–DC–T cell inflammatory cascade via inducing KC pyroptosis, thereby enhancing T cell activation and IL-17 production in psoriasis. Our study identifies that epidermal MHC-II promotes CXCL10-mediated NK cell infiltration. Subsequently, NK cells secrete GZMB, which activates GSDME and triggers KC pyroptosis to amplify psoriatic inflammation. These findings highlight the therapeutic potential of targeting MHC-II, NK cells or GSDME in psoriasis.

## Supplementary information

Supplementary Information

## Data Availability

The raw data of scRNA-seq and spatial transcriptomics for IMQ-treated skin from *H2-Ab1*^*fl/fl*^ mice and *K14.H2-Ab1*^*fl/fl*^ mice have been deposited in the NCBI SRA database under the accession numbers PRJNA1334276 and PRJNA1334280. The raw files of scRNA-seq for epidermal tissues from patients with psoriasis and HCs have been deposited in the National Omics Data Encyclopedia under the accession number OEP00006630. Code details are available upon reasonable request.
