# Supplementary material for: Epidermal MHC-II-mediated NK cell recruitment triggers keratinocyte pyroptosis, facilitating pathogenesis of psoriasis
Source: Exp Mol Med. 2026 May 8;58(5):1573–88. doi: 10.1038/s12276-026-01717-z (PMC13233949; doi:10.1038/s12276-026-01717-z)
Supplement: Supplementary file 1 — Supplementary Information [file 12276_2026_1717_MOESM1_ESM.pdf]

Supplementary Materials for

Epidermal MHC-II-mediated NK cell recruitment triggers keratinocyte pyroptosis facilitating pathogenesis of psoriasis

Authors: Xiaoqing Yi [1,2,3,4,5] [#], Pian Yu [1,2,3,4,5] [#], Jiayi Wang[1,2,3,4,5], Chi Fang[1,2,3,4,5], Sihui Ma[1,2,3,4,5], Kaixuan Li[1,2,3,4,5], Rongxuan Yan[1,2,3,4,5], Guanming Wang[1,2,3,4,5], Yihui Chen[1,2,3,4,5], Chao Chen[1,2,3,4,5], Detian Zhang[1,2,3,4,5], Yehong Kuang[1,2,3,4,5], Wu Zhu[1,2,3,4,5], Jie Li [1,2,3,4,5] , Guoqiang Zhang[6] 🖂, Tuo Deng[7,8,9]🖂, Xiang Chen[1,2,3,4,5] 🖂, Cong Peng[1,2,3,4,5] 🖂

# These authors contributed equally to this work.

🖂 Correspondence: **pengcongxy@csu.edu.cn.**

This file includes:

Supplementary Fig. 1-19

Supplementary Table 1-2

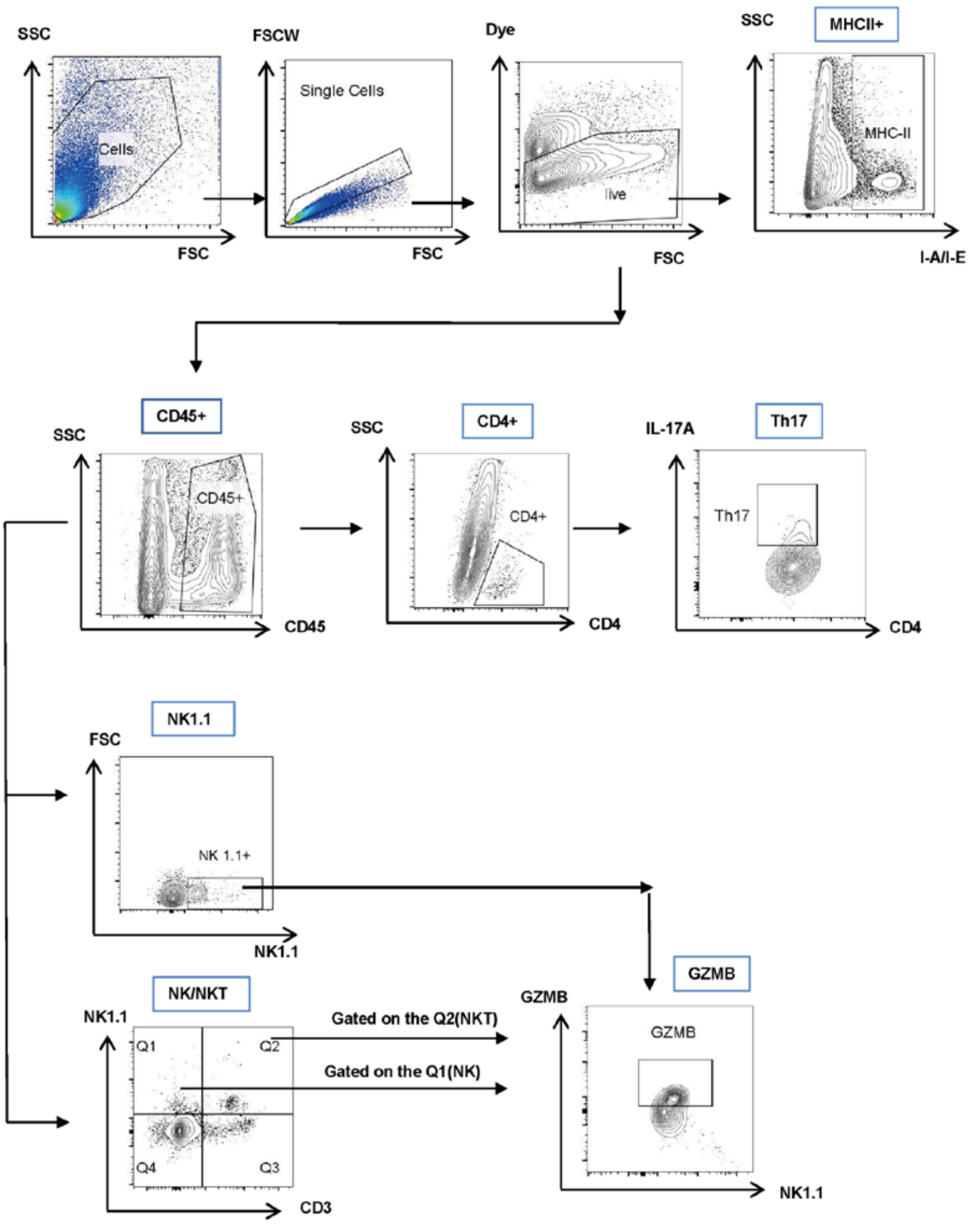

Supplementary Fig. 1 The representative flow diagram of gating strategies for Th17, NK, NKT cells, I-A/I-E+ cells in the spleen or skin tissues.

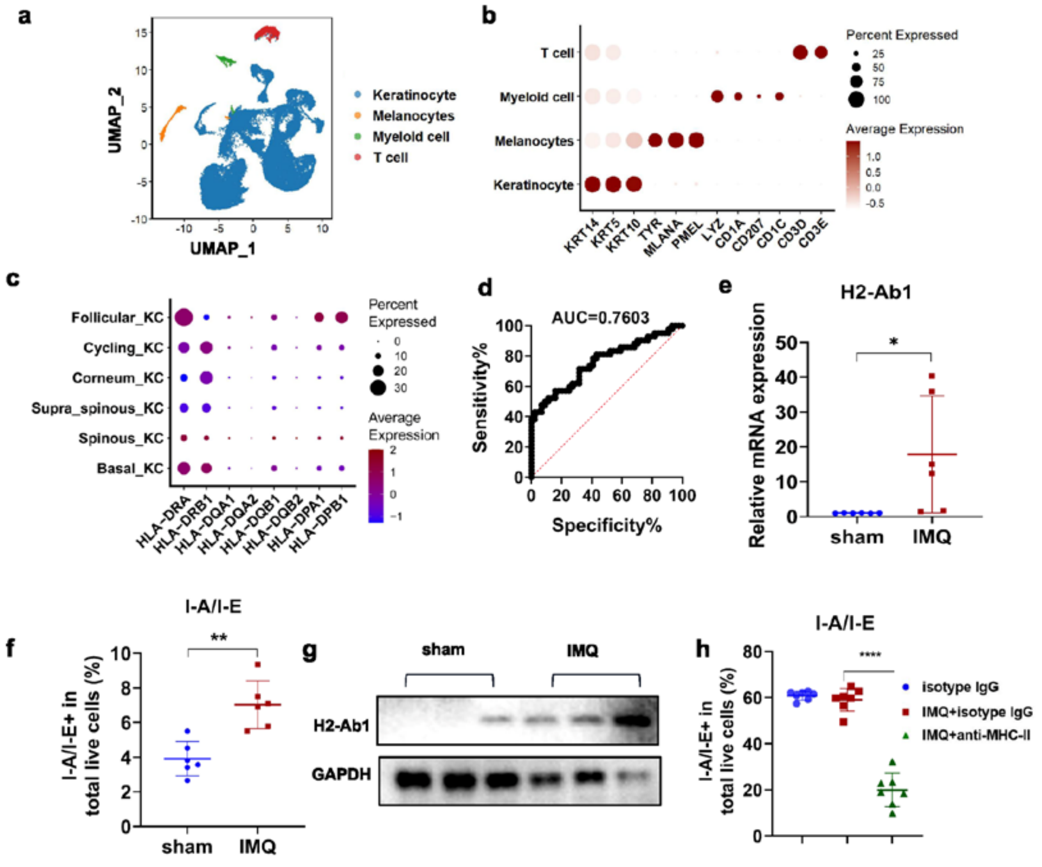

Supplementary Fig. 2 Anti-MHC-II antibody application relieves the phenotypes of IMQ-induced mice. a UMAP plotting of cell clusters in epidermal tissues. **b** The dot plot showing the representative markers of each cell cluster. **c** The dot plot showed the expression distribution of MHC-II genes in different keratinocyte (KC) clusters. **a-c** based on the scRNA-seq analysis of epidermal samples from psoriasis patients and healthy control. **d** The diagnostic value of MHC-II expressions in the epidermis for psoriasis was calculated by receiver operating characteristic (ROC) curve analysis (Healthy control, HC; psoriasis, Pso). **e-g** Increased MHC-II expression in the skin derived from sham or IMQ-treated mice by RT-PCR (**e**), flow cytometry (**f**) and WB (**g**). n = 3 or 6. **h** Flow cytometric analysis of the I-A/I-E percentage gated on the total live cells in the spleen of IMQ-treated mice after the anti-MHC-II application. n = 7.

Data were presented as mean±SD. *P* values were determined using one-way ANOVA with Tukey' s multiple comparisons test or two-tailed Student's t test. *$p < 0.05$, **$p < 0.01$, ***$p < 0.001$, ****$p < 0.0001$.

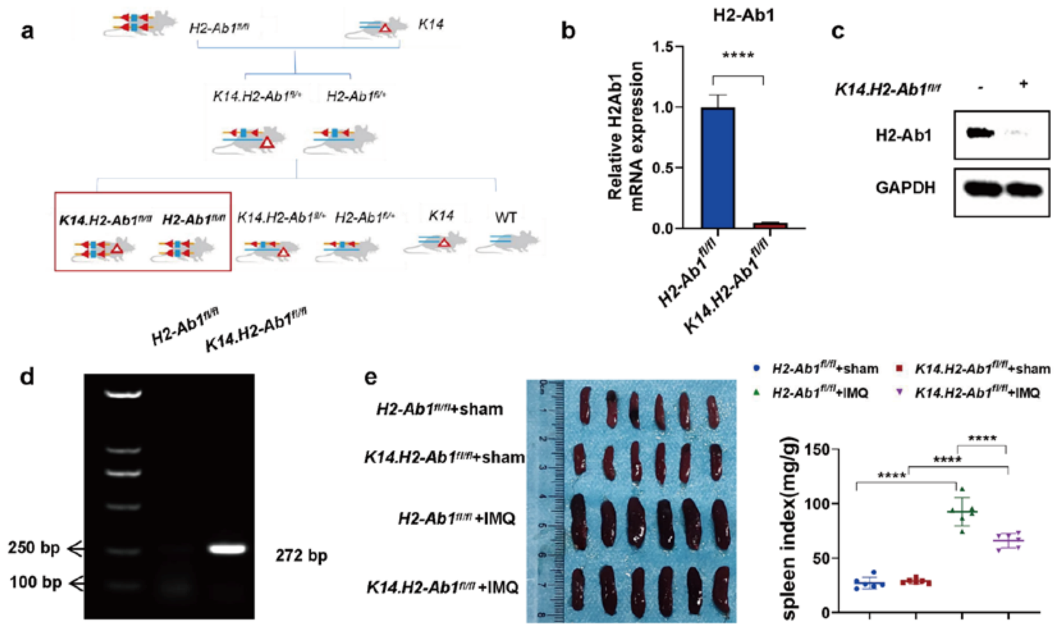

Supplementary Fig. 3 Keratinocyte-specific H2-Ab1 knockout mitigates the psoriasis-like inflammation. a Schematic representation of the generation of the *H2-Ab1*$^{fl/fl}$ and *K14.H2-Ab1*$^{fl/fl}$ mice. **b, c** The H2-Ab1 mRNA (**b**) and protein (**c**) levels in the epidermis of *H2-Ab1*$^{fl/fl}$ and *K14.H2-Ab1*$^{fl/fl}$ mice. n = 3. **d** Excision of floxed H2-Ab1 alleles was confirmed in K14-Cre recombinase transgenic mice by genotyping. **e** The size of spleens and statistical analysis of the spleen index (mg/g) of keratinocyte-specific H2-Ab1 knockout mice and the control mice with or without IMQ application. n = 6. Data were presented as mean±SD. *P* values were determined using one-way ANOVA with Tukey's multiple comparisons test or two-tailed Student's t test. ****$p < 0.0001$.

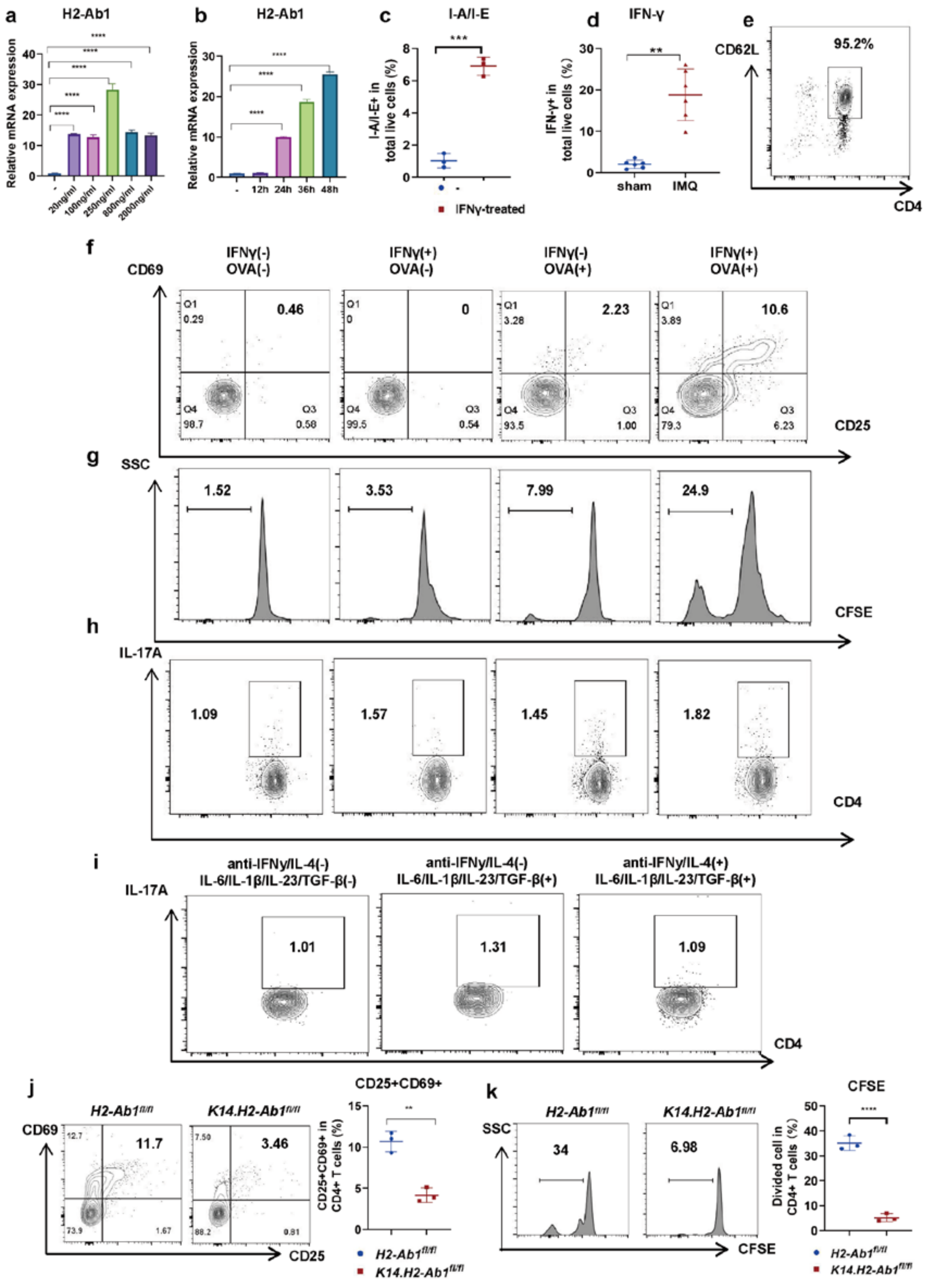

Supplementary Fig. 4 Keratinocyte-specific H2-Ab1 knockout impairs antigen-dependent CD4$^+$ T cell activation and proliferation. a, b H2-Ab1 mRNA expression of KCs stimulated by IFN-γ (250 ng/ml) with different concentrations (**a**) or time (**b**). **c** Increased MHC-II expression in KCs after IFN-γ treatment by flow cytometry. **d**

Increased IFN-γ percentage gated on the total live cells in the cell suspension from the skin with IMQ topical application. **e** The purity of the enriched naïve CD4$^+$ T cells from the spleen characterized as the percentage of CD4$^+$CD62L$^+$ cell subset was generally higher than 95% validated by flow cytometry. **f-h** Naïve CD4$^+$ T cells were cocultured with untreated or IFN-γ-pretreated KCs loaded with OVA or not, and then the levels of activation (**f**), proliferation (**g**) and the differentiation into Th17 cells (**h**) of naïve CD4$^+$ T cells were analyzed by flow cytometry. **i** Naïve CD4$^+$ T cells failed to differentiate into Th17 cells when cocultured with IFN-γ and OVA-pretreated KCs plus anti-IFN-γ/IL-4 or IL-6/IL-1β/IL-23/TGF-β. **j, k** Representative flow diagram and statistical analysis of the activation (**j**) and proliferation (**k**) levels of naïve CD4$^+$ T cells cocultured with the H2-Ab1-deficient KCs under the IFN-γ and OVA-stimulation. Data were presented as mean ± SD. *P* values were determined using one-way ANOVA with Tukey's multiple comparisons test or two-tailed Student's t test. **p* < 0.05, ***p* < 0.01, ****p* < 0.001, *****p* < 0.0001.

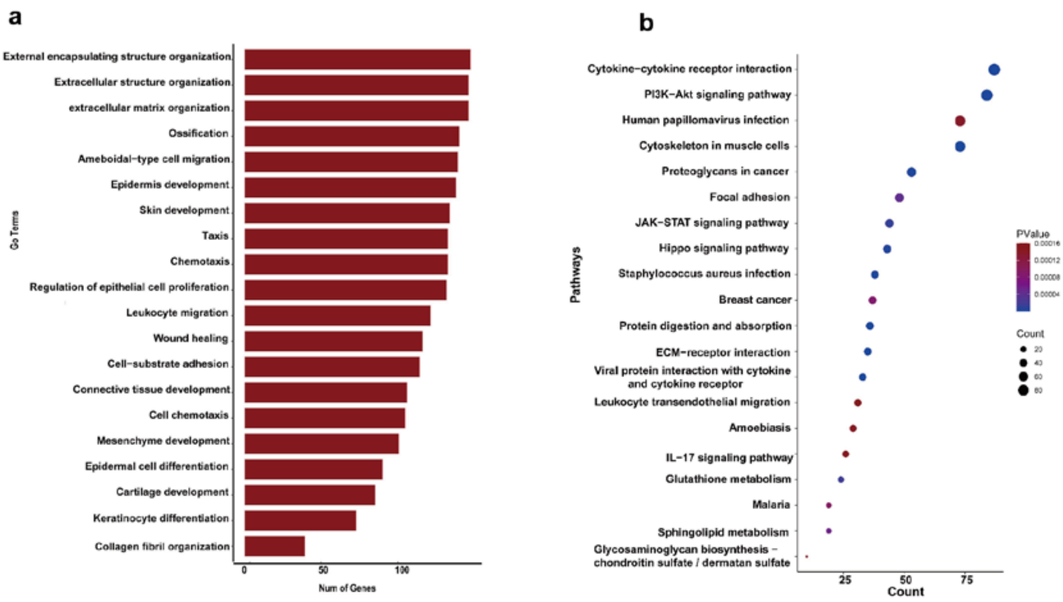

Supplementary Fig. 5 H2-Ab1 deletion in keratinocytes affects the functional pathways of the skin under IMQ topical application. a, b Enriched Gene Ontology (GO) terms (**a**) and KEGG pathways (**b**) of DEGs in the skin from the *H2-Ab1*$^{fl/fl}$ and *K14.H2-Ab1*$^{fl/fl}$ mice with IMQ topical application identified by scRNA-seq.

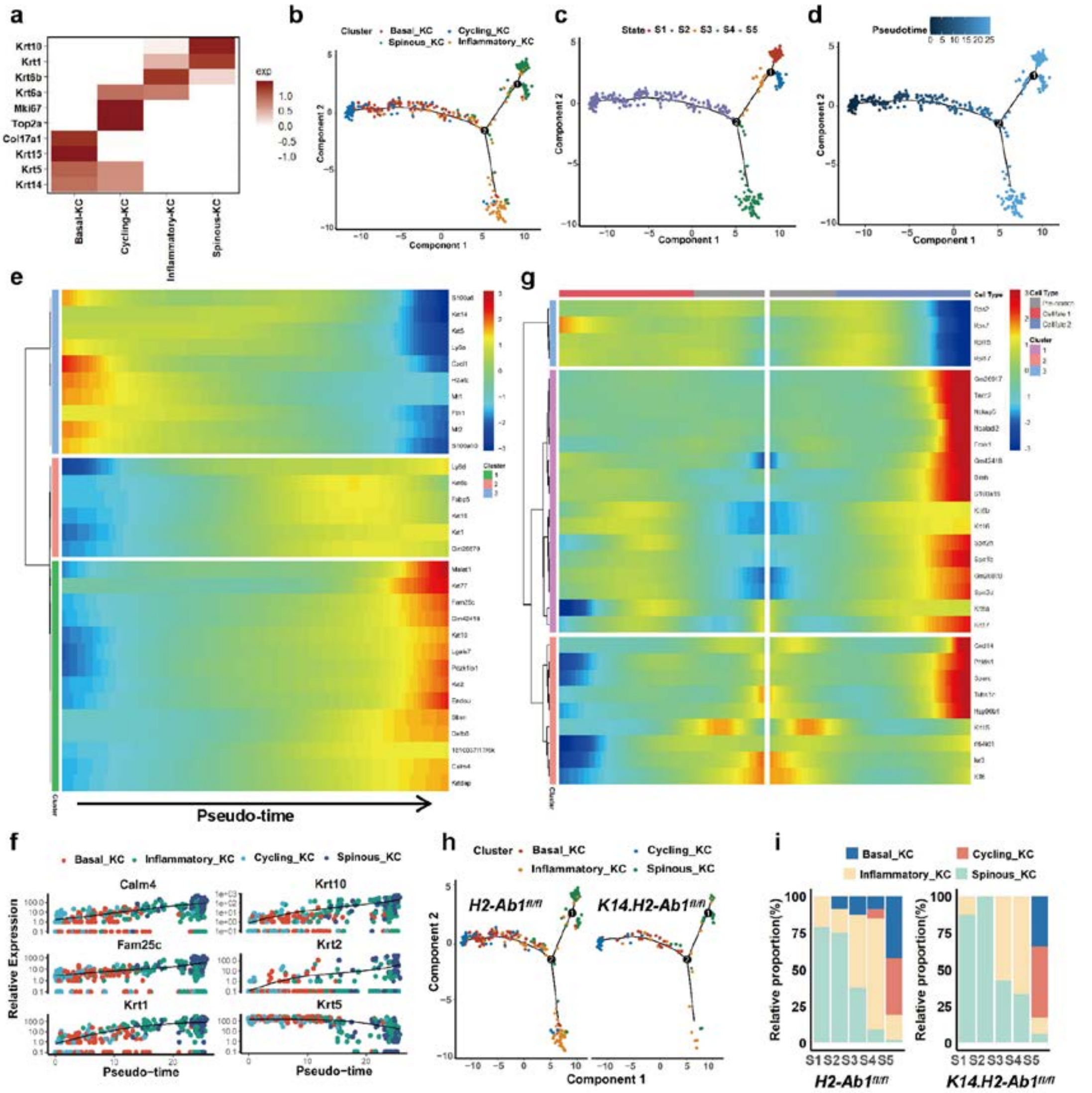

Supplementary Fig. 6 Cell composition and pseudotime analysis of keratinocytes. a Levels and distributions of selected genes for each subpopulation in KCs visualized in the form of a heatmap. **b-d** Unsupervised transcriptional trajectory of KCs from Monocle2, colored by cell subsets (**b**), cell states (**c**) or pseudotime (**d**). **e** The

expression heatmap of top 30 differential genes along with pseudotime. These signature genes were divided into 3 clusters based on their expression trend. **f** Expressions of top 6 differential genes over pseudotime trajectory map. **g** The branched expression heatmap of top 30 differential genes along with pseudotime at branch point 1 for different cell fates. **h** Cell trajectory plot of KCs respectively for *H2-Ab1*$^{fl/fl}$ and *K14.H2-Ab1*$^{fl/fl}$ mice. **i** Relative proportion of cell subsets for each cell state in *H2-Ab1*$^{fl/fl}$ and *K14.H2-Ab1*$^{fl/fl}$ mice.

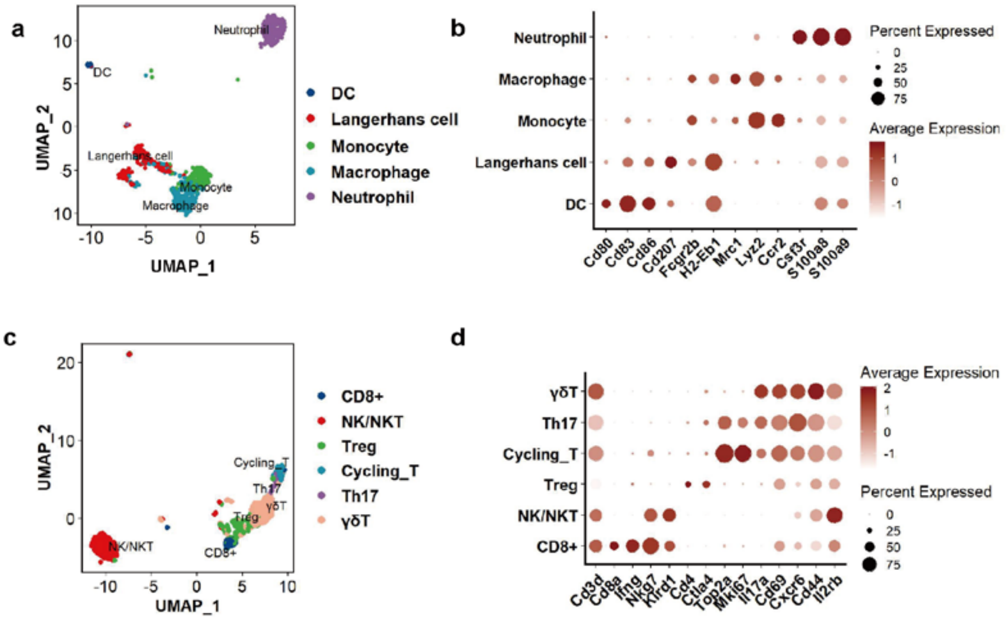

Supplementary Fig. 7 Cell subset identification of immune cells. a UMAP plotting of cell subpopulations of myeloid cells. **b** The dot plot showing the representative markers of myeloid cells. **c** UMAP plotting of cell subpopulations of T cells. **d** The dot plot showing the representative markers of T cells.

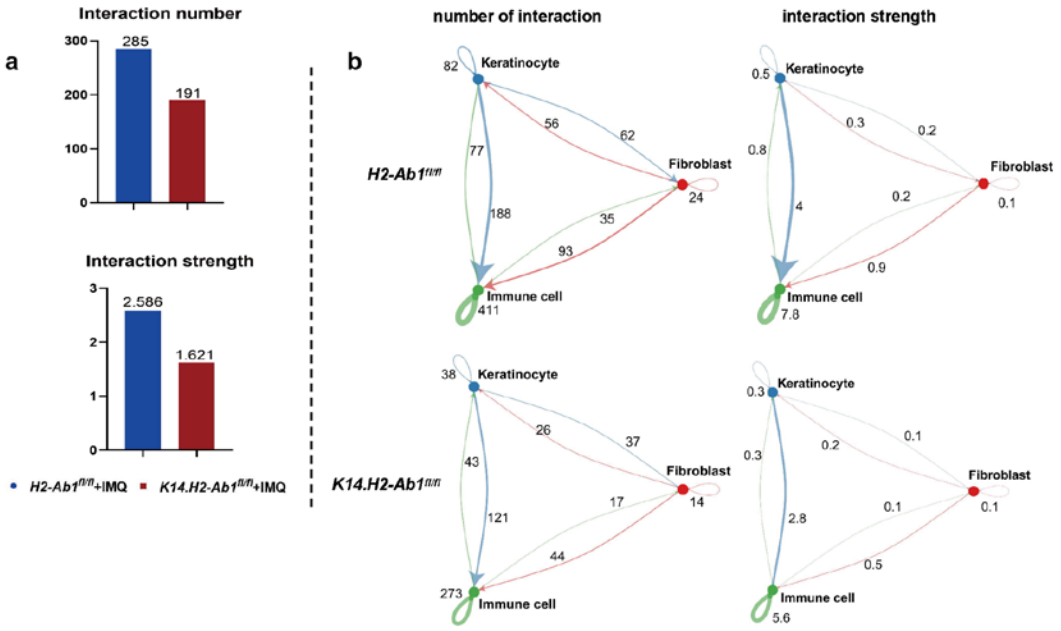

Supplementary Fig. 8 Ligand-receptor interactions analysis by CellChat. a Total interaction number or strength among major cell types by CellChat. **b** The number or strength of ligand-receptor pairs between two cell populations among KCs, fibroblasts and immune cells in *H2-Ab1*$^{fl/fl}$ or *K14.H2-Ab1*$^{fl/fl}$ mice.

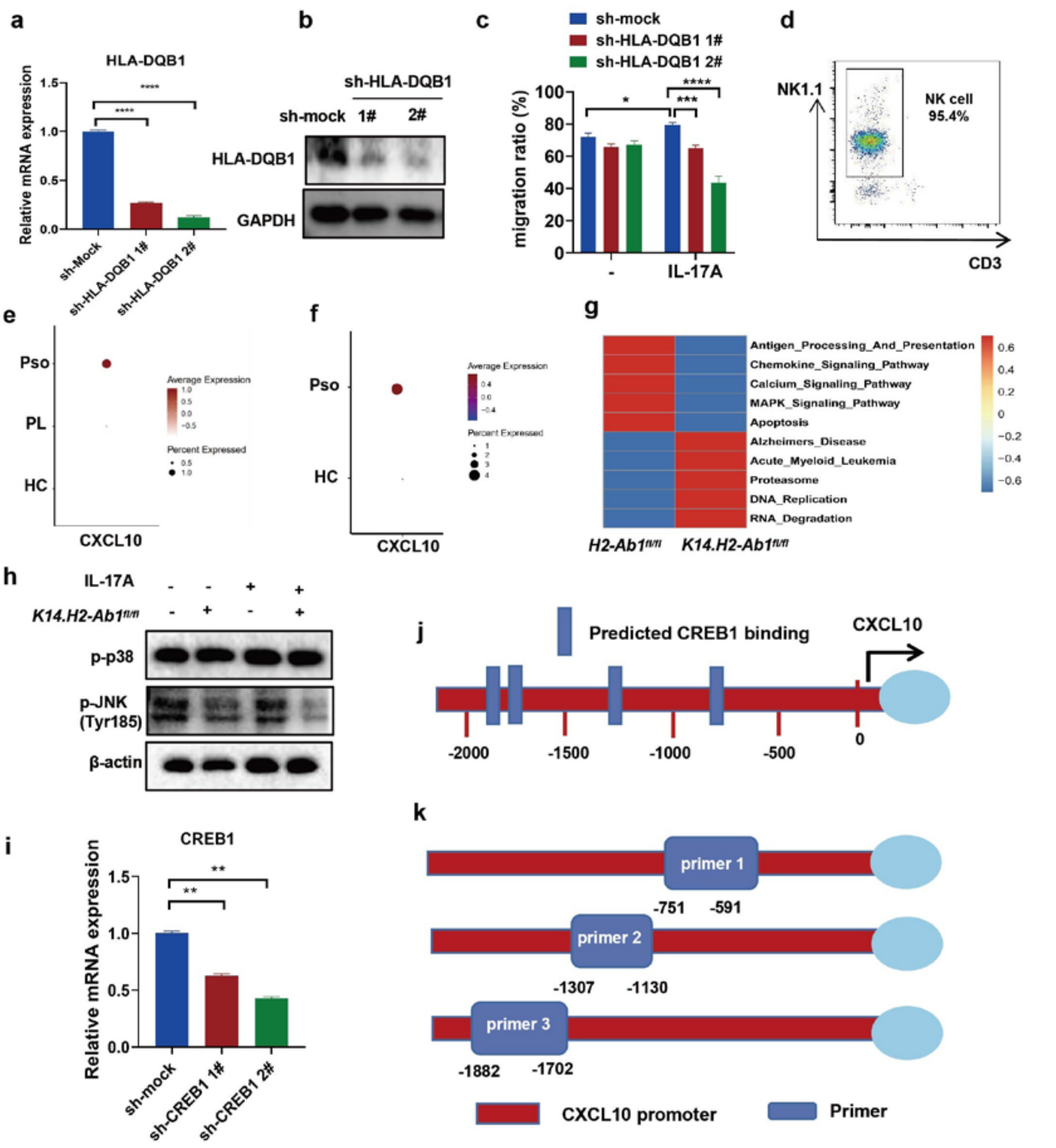

Supplementary Fig. 9 HLA-DQB1 in HaCaTs influences the CREB/CXCL10 axis. **a, b** HaCaTs were transfected with vector or HLA-DQB1 plasmid. The HLA-DQB1 mRNA (**a**) and protein levels (**b**) were detected. **c** HaCaTs were infected with vector or HLA-DQB1 lentivirus and then treated with or without IL-17A for 24h. The migration ratio of NK92 was calculated after co-culture with HaCaTs for 24h. **d** The purity of the enriched NK cells from the spleen (Flow analysis marker: $CD45^{+}CD3^{-}NK1.1^{+}$). **e** The CXCL10 expression of the KC cluster in the form of dot plot under different skin

conditions based on datasets GSE162183 and GSE173706 (healthy skin [HC], paralesional skin [PL], psoriatic skin [Pso]). **f** The CXCL10 expression in the form of dot plot based on our scRNA-seq data from epidermal tissues of psoriatic patients (Pso) and healthy control (HC). **g** Heatmap of enriched KEGG pathways across differentiated genes in KCs using the GSVA based on the scRNA-seq analysis of IMQ-treated skin. **h** Western blot analysis of p-p38 and p-JNK expressions in KCs isolated from the newborn *H2-Ab1*$^{fl/fl}$ mice and *K14.H2-Ab1*$^{fl/fl}$ mice with or without IL-17A (100 ng/ml) stimulation for 30 min. **i** The CREB1 mRNA expression in HaCaTs transfected with vector or CREB1 plasmid. **j** CREB1 binding sites on the promoter region of CXCL10 predicted by the Cistrome Data Browser and JASPAR Database. **k** Designed primers for ChIP experiments. Data in (**a-c, h, i**) were representative of three independent experiments. Data were shown as the mean ± SD. *P* values were determined using one-way ANOVA with Tukey's multiple comparisons test. *$p < 0.05$, **$p < 0.01$, ***$p < 0.001$, ****$p < 0.0001$.

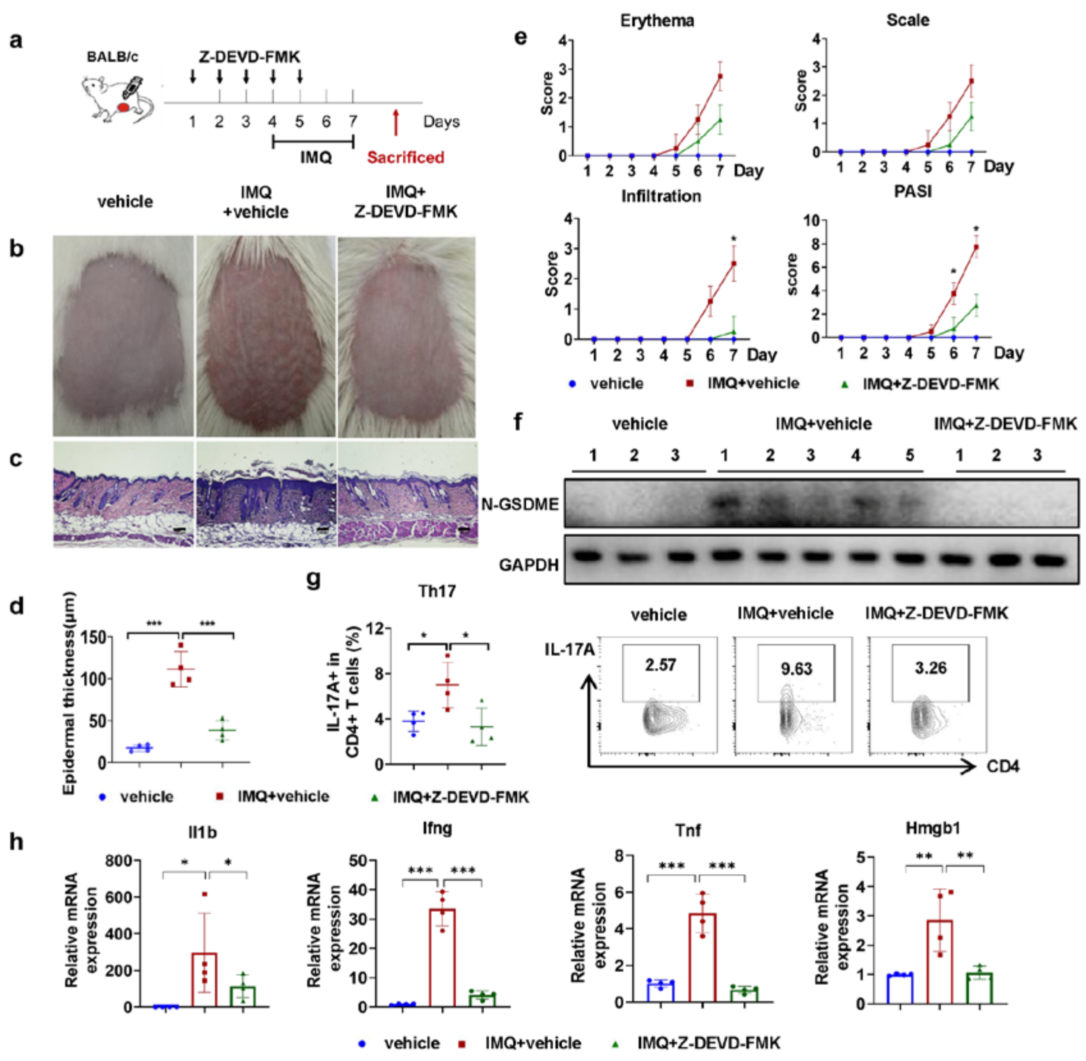

Supplementary Fig. 10 Inhibition of Gsdme activation blocks psoriasis-like inflammation. a Schematic diagram of Z-DEVD-FMK intraperitoneal administration (200 mg/kg) in mice with IMQ topical application. **b-e** Phenotypic presentation (**b**) and H&E staining (**c**) as well as statistical analysis of the epidermal thickness (**d**) and PASI scores (**e**) of back skin in control or IMQ-treated mice injected with vehicle or Z-DEVD-FMK. Scale bars: 100 μm. **f** Protein was extracted from the back skin of the control or IMQ-treated mice injected with vehicle or Z-DEVD-FMK, and immunoblotting was then performed as indicated. The expression of GAPDH served as a protein loading control. **g** Flow cytometric plots and quantification of the Th17 percentage gated on $CD4^+$ T cells as indicated. **h** The mRNA expression of pyroptosis-related factors in the back skin as indicated. n = 4. Data were shown as the mean ±

SD. *P* values were determined using one-way ANOVA with Tukey' s multiple comparisons test or two-tailed Student's t test. *$p < 0.05$, **$p < 0.01$, ***$p < 0.001$.

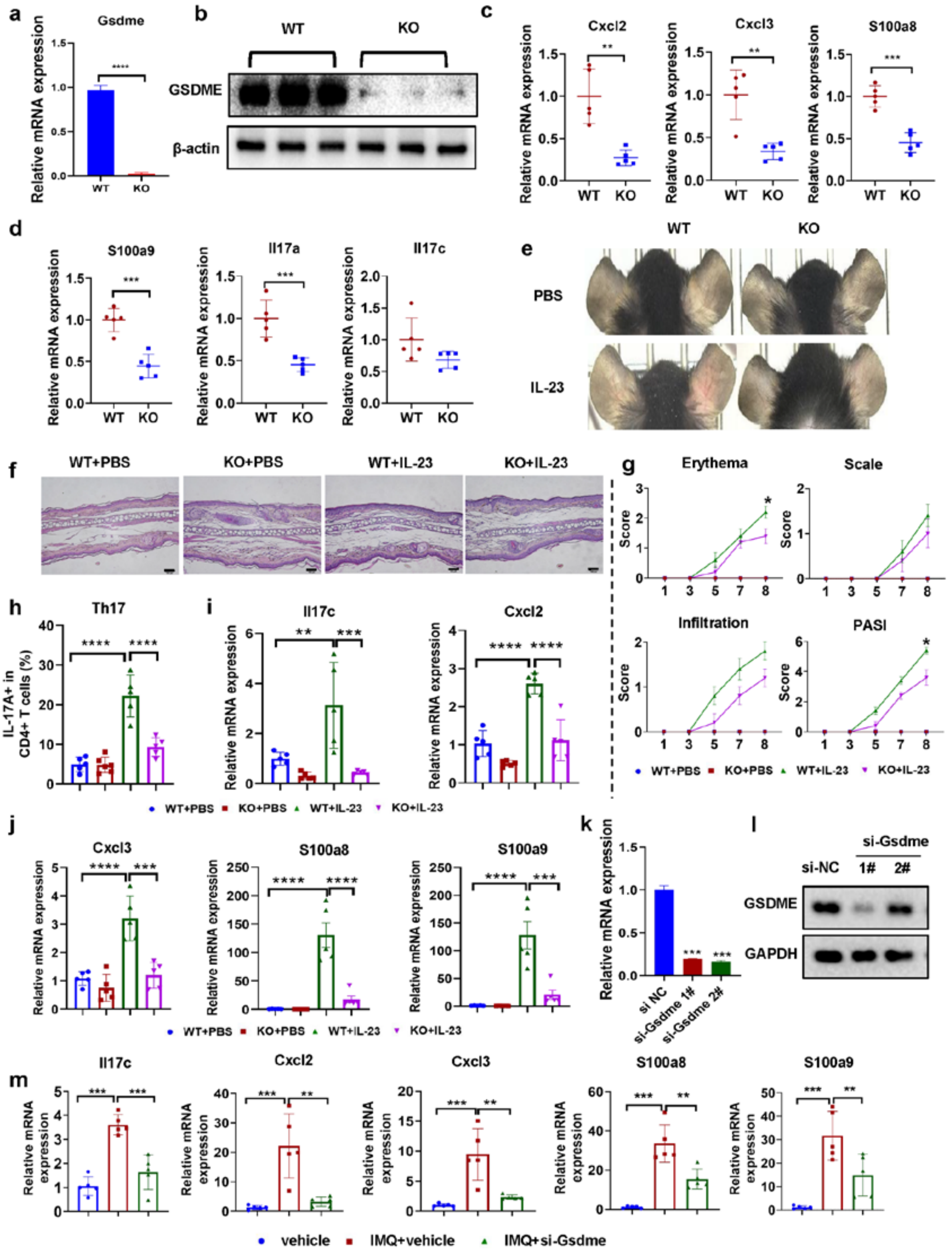

Supplementary Fig. 11 Targeting Gsdme attenuates IMQ-induced psoriatic inflammation. a, b The mRNA (**a**) and protein (**b**) levels of Gsdme in skin samples of

WT and *Gsdme*$^{-/-}$ mice. **c, d** Transcription levels of Il17a, Il17c, Cxcl2, Cxcl3, S100a8, S100a9 in the skin of *Gsdme*$^{-/-}$ and WT mice treated with IMQ. **e-g** Phenotypic presentation (**e**) and H&E staining (**f**) as well as PASI scores (**g**) of the ear skin in WT and *Gsdme* KO mice subcutaneous injected with PBS or IL-23. Scale bars: 100 μm. **h** Quantification of the Th17 cell percentage gated on the CD4^{+} T cells as indicated. **i, j** The mRNA expression of psoriasis-related factors in the skin as indicated. **k, l** Primary KCs were transfected with the siRNA against Gsdme (si-Gsdme) or a control siRNA (si-NC). The Gsdme mRNA (**k**) and protein levels (**l**) were detected. **m** Transcription levels of Il17c, Cxcl2, Cxcl3, S100a8, S100a9 in the back skin of control or IMQ-treated mice with or without si-Gsdme topical application. n = 5. Data were shown as the mean ± SD. *P* values were determined using one-way ANOVA with Tukey's multiple comparisons test or two-tailed Student's t test. $*p < 0.05$, $**p < 0.01$, $***p < 0.001$, $****p < 0.0001$.

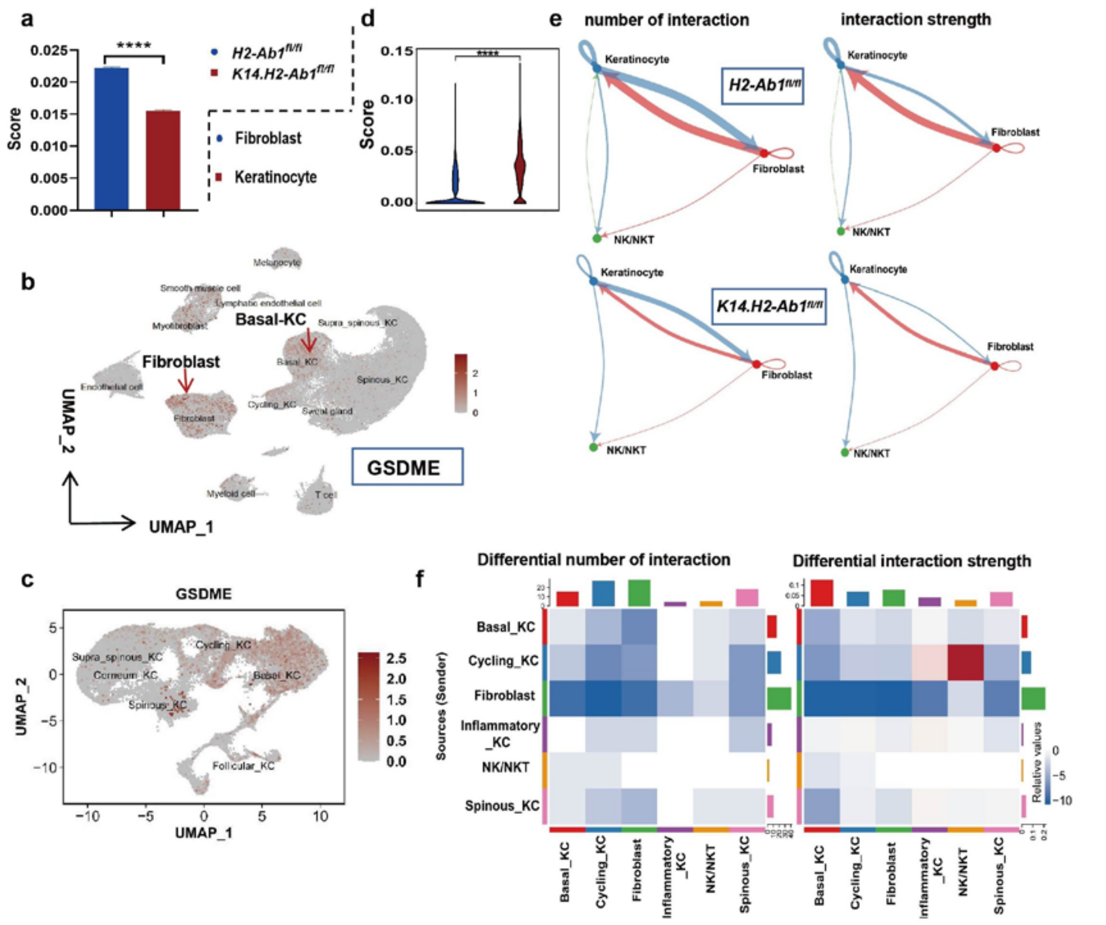

Supplementary Fig. 12 NK cells predominantly target keratinocytes to induce Gsdme-mediated pyroptosis. a Statistical analysis of the differences in Ucell scores for pyroptosis-related gene signatures between *H2-Ab1*$^{fl/fl}$ and *K14.H2-Ab1*$^{fl/fl}$ mice. **b** The GSDME expression at different skin cell types projected on the UMAP plot (datasets GSE162183 and GSE173706). **c** The GSDME expression at different KC clusters in epidermal tissues from psoriasis patients (Pso) and healthy control (HC) based on our scRNA-seq data. **d** Violin plots exhibiting the differences in Ucell scores for pyroptosis-related gene signatures between KCs and fibroblasts**. e** The number or strength of ligand-receptor pairs between two cell populations among keratinocytes, fibroblasts and NK/NKT cells in *H2-Ab1*$^{fl/fl}$ and *K14.H2-Ab1*$^{fl/fl}$ mice based on scRNA-

seq results. **f** The differential interaction number or strength between KC subclusters and NK/NKT cells. The color on the heatmap indicated the predominance of *H2-Ab1*$^{fl/fl}$ or *K14.H2-Ab1*$^{fl/fl}$ mice in the interaction number or strength for each cell type pair (blue: *H2Ab1*$^{fl/fl}$; red: *K14.H2Ab1*$^{fl/fl}$) based on scRNA-seq results.

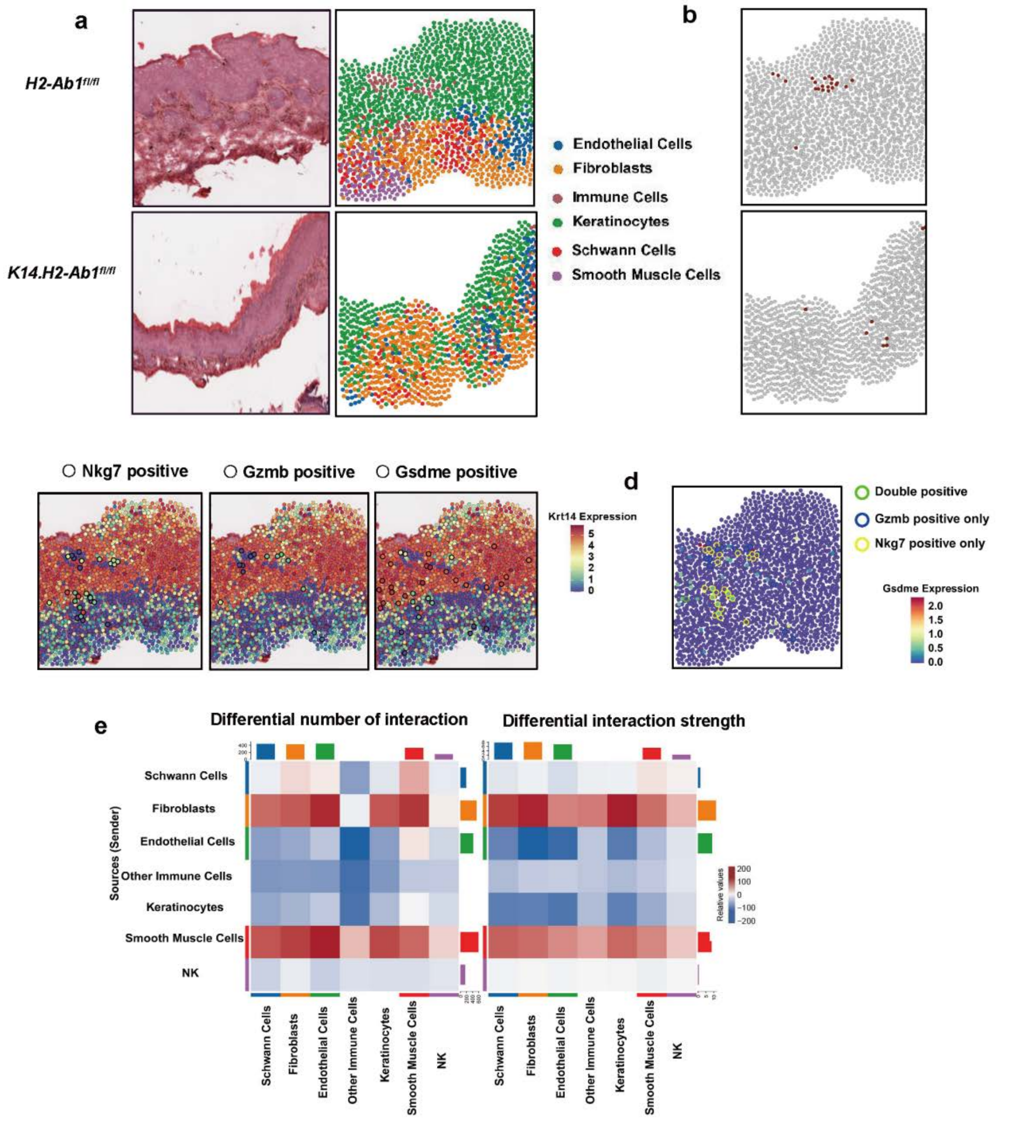

Supplementary Fig. 13 Spatial orientation and interaction of NK cells and keratinocytes. a Integration of scRNA-seq and spatial transcriptomics data for the IMQ-treated mouse ear skin cell type identification. Representative H&E image and corresponding spatial feature plots of predicted cell type were displayed. **b**

Corresponding spatial feature plots of NK cells. **c** Spatial gene expression plots of Nkg7, Gzmb and Gsdme in the epidermis (marker: Krt14) of IMQ-induced psoriasis-like skin. **d** Spatial mapping of the expression and co-localization of Nkg7 and Gzmb in IMQ-induced psoriasis-like skin. **e** The differential interaction number or strength among skin major cell types and NK cells based on the spatial transcriptomic results of IMQ-treated skin. The color on the heatmap indicated the predominance of *H2-Ab1*$^{fl/fl}$ or *K14.H2-Ab1*$^{fl/fl}$ mice in the interaction number or strength for each cell type pair (blue: *H2-Ab1*$^{fl/fl}$; red: *K14.H2-Ab1*$^{fl/fl}$).

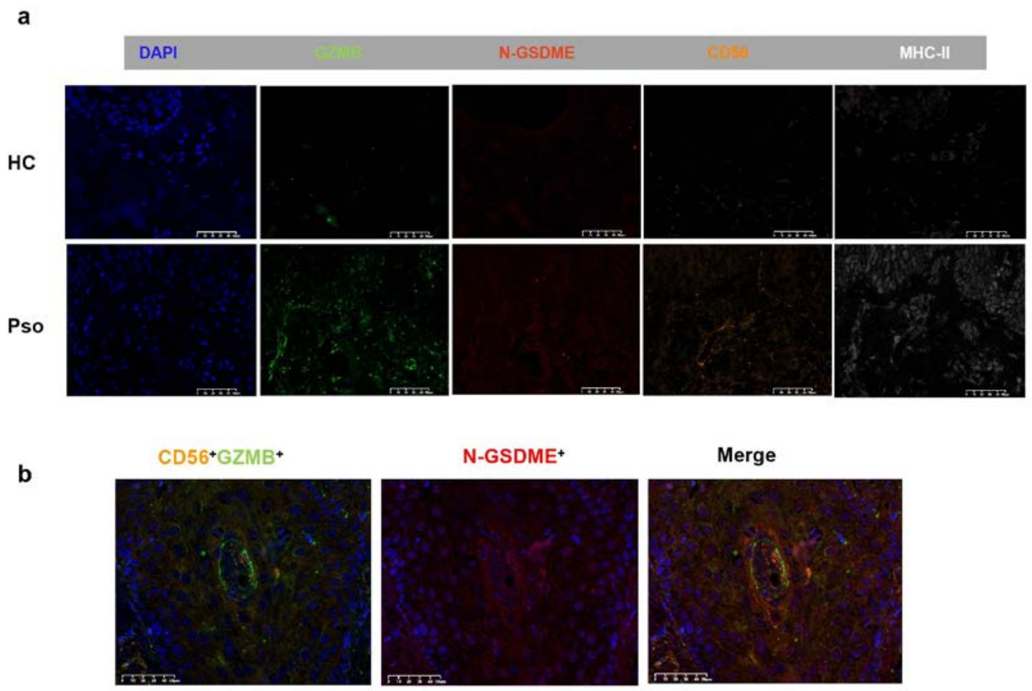

Supplementary Fig. 14 Multiplex immunohistochemistry reveals NK cell-mediated keratinocyte pyroptosis in psoriasis. **a** Representative multiple immunohistochemical staining images of MHC-II, CD56, GZMB and N-GSDME in the skin of healthy control (HC) and psoriasis (Pso) at 400x magnification. **b** The colocalization of CD56^{+}GZMB^{+} NK cells with N-GSDME^{+} cells in the epidermis.

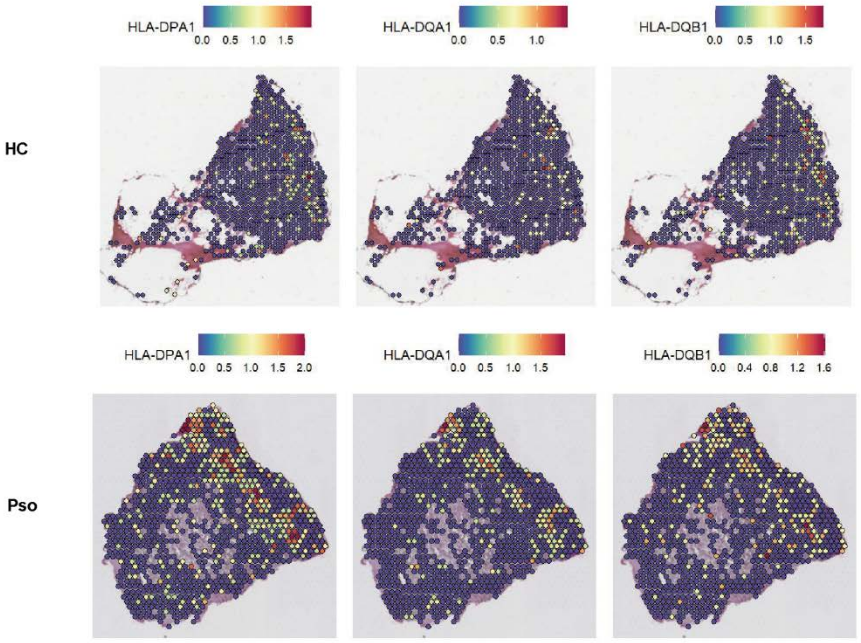

Supplementary Fig. 15 Spatial gene expression plots of HLA-DPA1, HLA-DQA1 and HLA-DQB1 in skin samples from HC and Pso patients based on the spatial transcriptomic data from GSE202011.

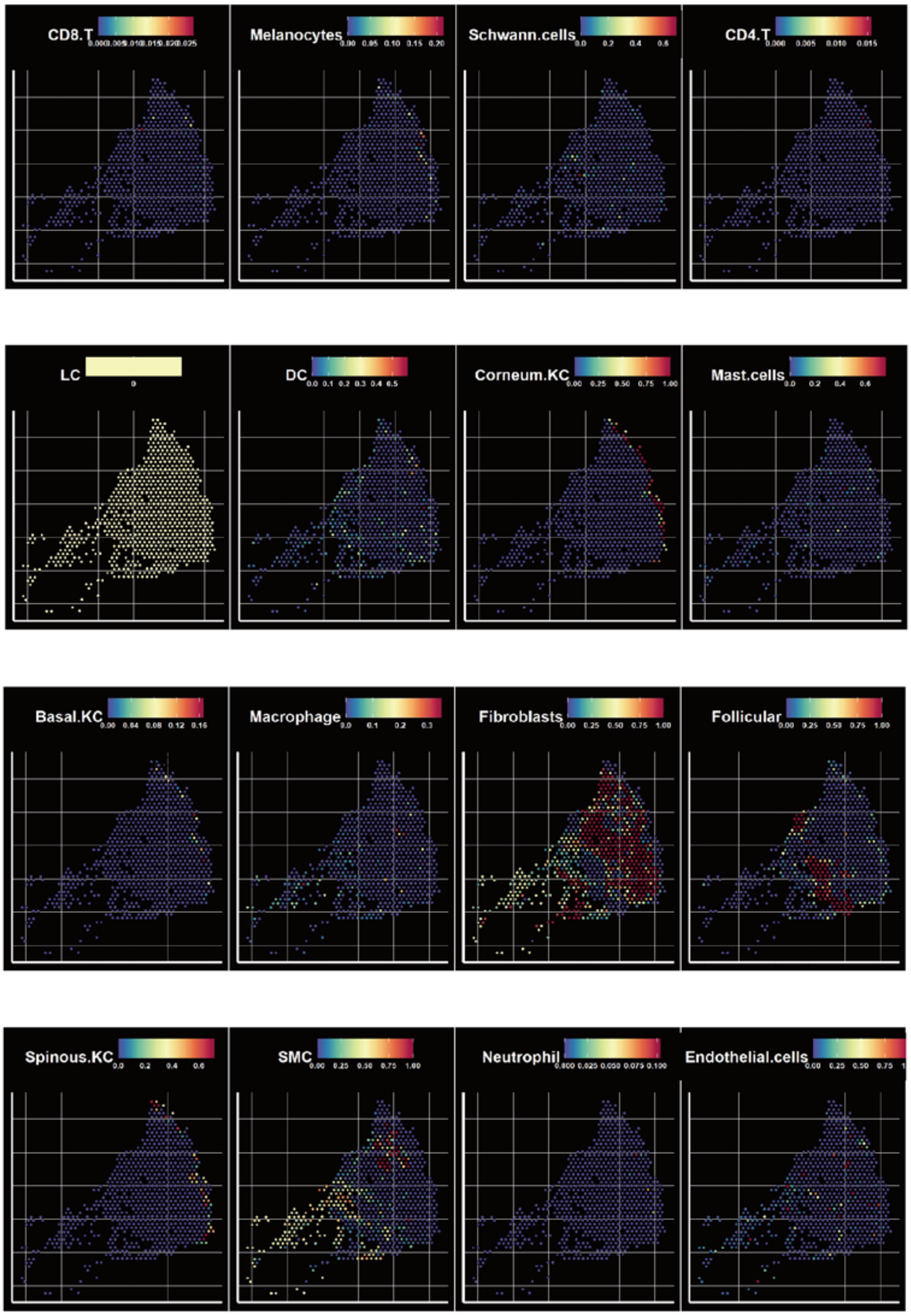

Supplementary Fig. 16 Corresponding spatial distribution for each cell type in skin samples from healthy control (HC) based on the spatial transcriptomic data from GSE202011. Analysis was performed by reference-based mapping against a scRNA-seq dataset (GSE173706).

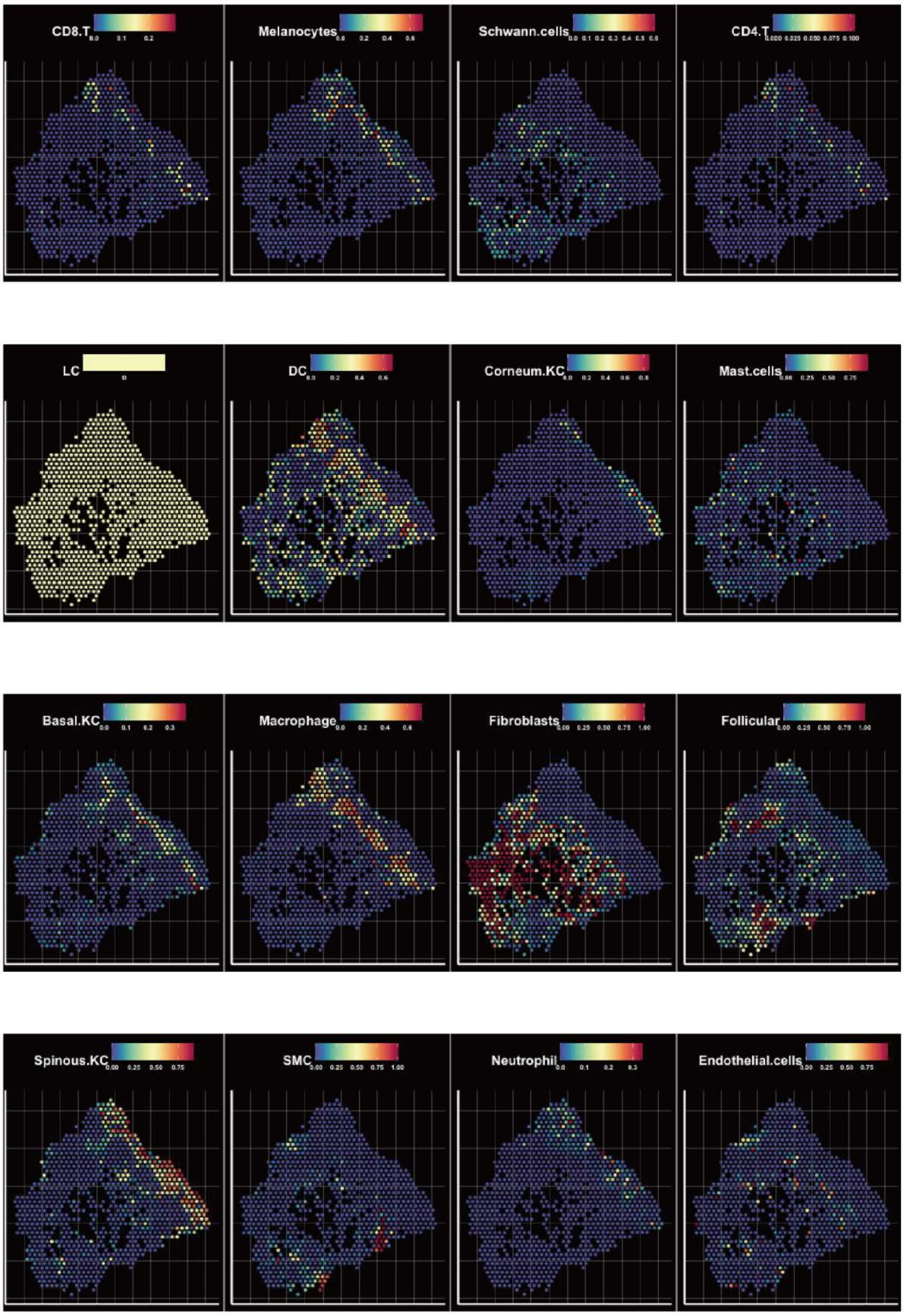

Supplementary Fig. 17 Corresponding spatial distribution for each cell type in psoriatic skin inferred from spatial transcriptomics data (GSE202011). Analysis was performed by reference-based mapping against a scRNA-seq dataset

(GSE173706).

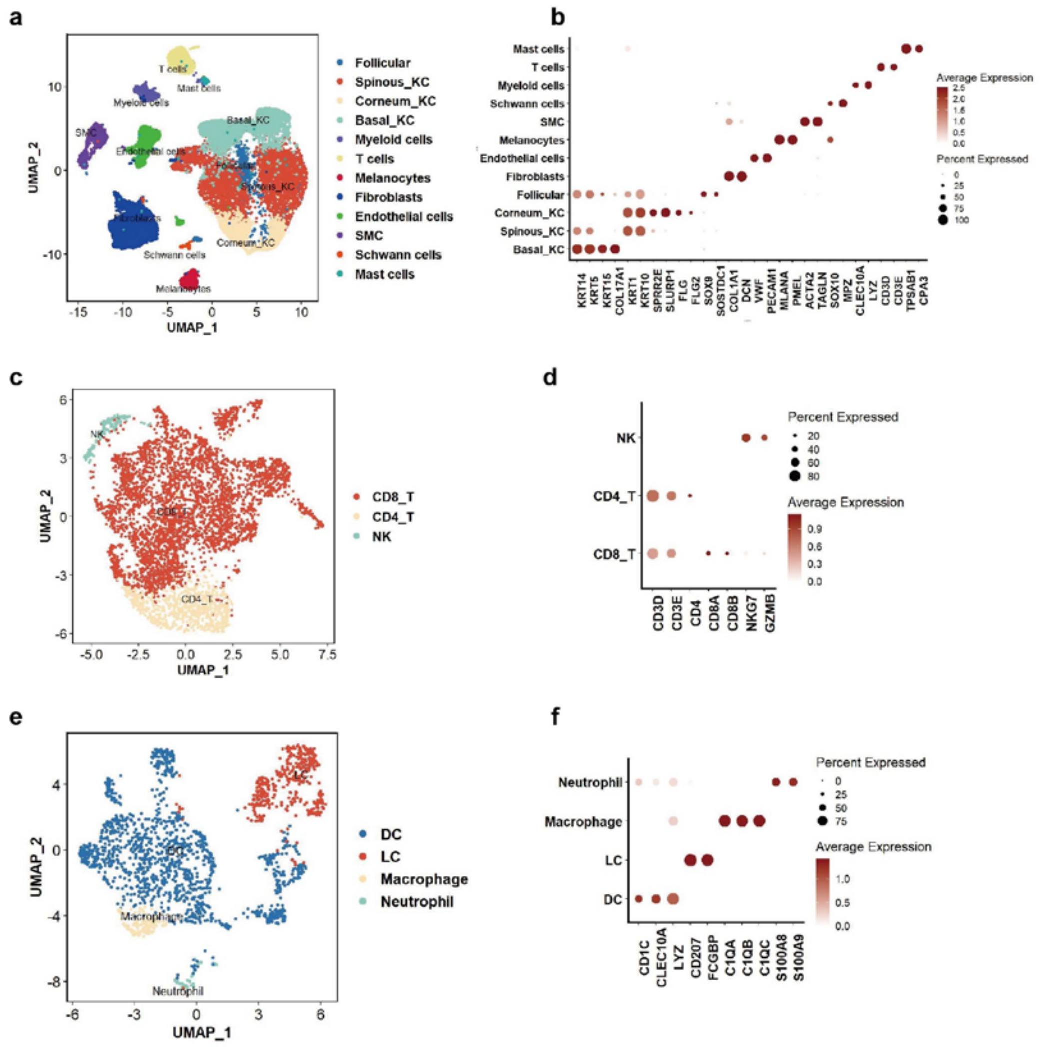

Supplementary Fig. 18 Cell subset identification analysis of scRNA-seq dataset GSE173706. a UMAP plotting of major cell populations. **b** The dot plot showing the representative markers of major cell populations. **c** UMAP plotting of cell subpopulations of T cells. **d** The dot plot showing the representative markers of T cells. **e** UMAP plotting of cell subpopulations of myeloid cells. **f** The dot plot showing the representative markers of myeloid cells. (SMC: Smooth Muscle Cell; LC: Langerhans Cell)

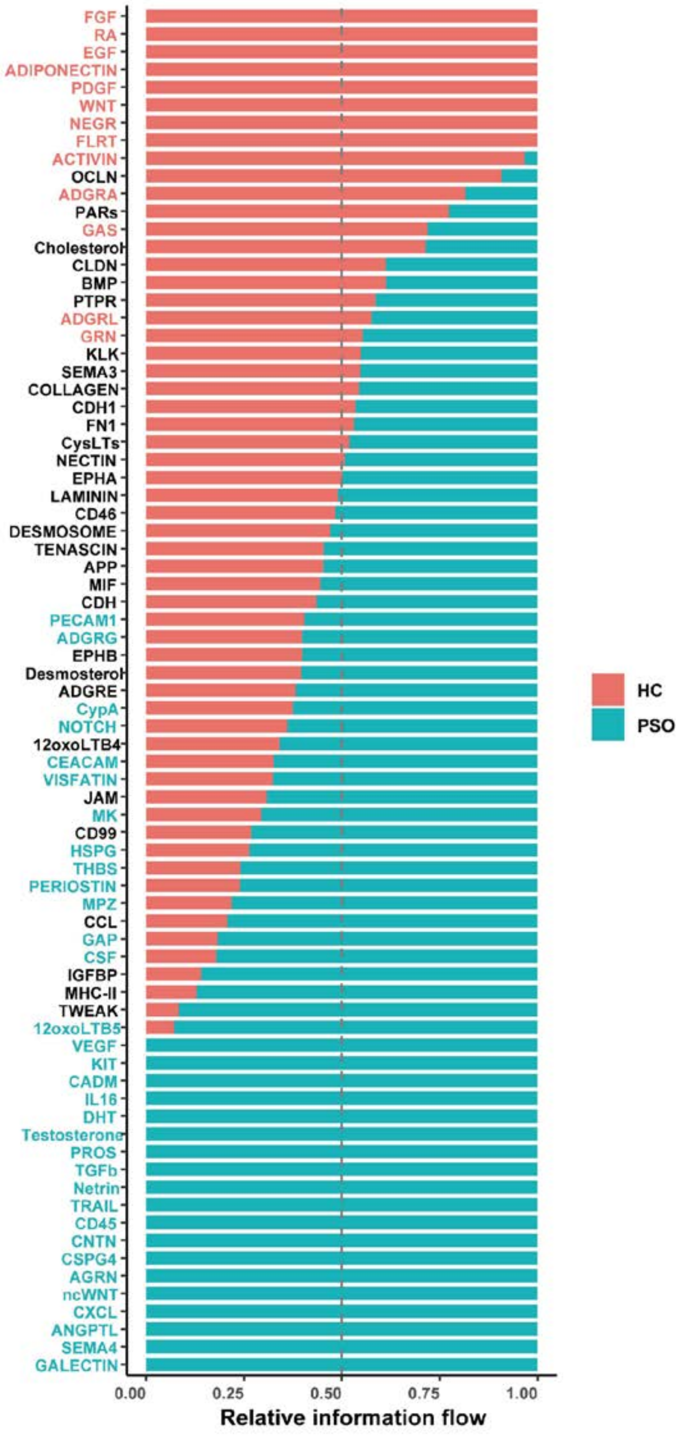

Supplementary Fig. 19 The comparison of the signaling pathway based on the relative information flow between HC and Pso based on the spatial transcriptomic data from GSE202011.

Supplementary Table 1 Demographic characteristics of psoriasis patients and healthy controls.

Healthy Control ID	Gender	Age	Psoriasis Patient ID	Gender	Age
HC-1	Female	21	PS-01	Female	31
HC-2	Female	30	PS-02	Male	45
HC-3	Female	21	PS-03	Female	26
HC-4	Female	19	PS-04	Male	26
HC-5	Female	20	PS-05	Female	75
HC-6	Female	25	PS-06	Female	56
HC-7	Female	42	PS-07	Female	16
HC-8	Female	49	PS-08	Female	14
HC-9	Female	52	PS-09	Female	30
HC-10	Female	52	PS-10	Female	46
HC-11	Female	63	PS-11	Male	63
HC-12	Female	65	PS-12	Male	21
HC-13	Female	66	PS-13	Male	47
HC-14	Female	67	PS-14	Male	51
HC-15	Female	49	PS-15	Female	55
HC-16	Female	72	PS-16	Female	69
HC-17	Female	77	PS-17	Male	18
HC-18	Female	25	PS-18	Male	47
HC-19	Male	19	PS-19	Female	32
HC-20	Male	23	PS-20	Male	28
HC-21	Male	13	PS-21	Female	22
HC-22	Male	32	PS-22	Female	49
HC-23	Male	24	PS-23	Male	33
HC-24	Male	26	PS-24	Male	26
HC-25	Male	21	PS-25	Female	61
HC-26	Male	44	PS-26	Male	53

HC-27	Male	46	PS-27	Male	53
HC-28	Male	53	PS-28	Female	48
HC-29	Male	58	PS-29	Male	59
HC-30	Male	60	PS-30	Male	19
HC-31	Male	30	PS-31	Female	59
HC-32	Male	61	PS-32	Female	69
HC-33	Male	42	PS-33	Male	57
HC-34	Male	62	PS-34	Male	30
HC-35	Male	63	PS-35	Male	33
HC-36	Male	63	PS-36	Male	18
HC-37	Male	47	PS-37	Female	34
HC-38	Male	69	PS-38	Female	29
HC-39	Male	75	PS-39	Male	16

Supplementary Table 2 Primer Sequences for quantitative real-time PCR

Primer	Forward (5'-3')	Reverse (5'-3')
Gsdme (M)	TGCAACTTCTAAGTCTGGTGACC	CTCCACAACCACTGGACTGAG
Gzmb (M)	CCACTCTCGACCCTACATGG	GGCCCCCAAAGTGACATTTATT
Ifng (M)	ATGAACGCTACACACTGCATC	CCATCCTTTTGCCAGTTCCTC
Tnf (M)	CCCTCACACTCAGATCATCTTCT	GCTACGACGTGGGCTACAG
Il1b (M)	GCAACTGTTCCTGAACTCAACT	ATCTTTTGGGGTCCGTCAACT
Hmgb1 (M)	GGCGAGCATCCTGGCTTATC	GGCTGCTTGTCATCTGCTG
H2-Ab1(M)	AGCCCCATCACTGTGGAGT	CTGTGTGGCAGCTGTGATCCT
Il17a (M)	TTTAACTCCCTTGGCGCAAAA	CTTTCCCTCCGCATTGACAC
Il17c (M)	CCTCTAGCTGGAACACAGTGC	GCGGTTCTCATCTGTGTCG
S100a8 (M)	TCCTTGCGATGGTGATAAAA	GGCCAGAAGCTCTGCTACTC
S100a9 (M)	CACAGTTGGCAACCTTTATGAA	TCATACACTCCTCAAAGCTCAG
Cxcl2 (M)	AAAATCATCCAAAAGATACTGAACAA	CTTTGGTTCTTCCGTTGAGG
Cxcl3 (M)	CCCCAGGCTTCAGATAATCA	TCTGATTTAGAATGCAGGTCCTT
Cxcl10(M)	CCAAGTGCTGCCGTCATTTTC	GGCTCGCAGGGATGATTTCAA
Actb (M)	TATGCTCTCCCTCACGCCATCC	GTCACGCACGATTTCCCTCTCAG
ACTB (H)	CCTGGCACCCAGCACAAT	GGGCCGGACTCGTCATAC
HLA-DQB1 (H)	ACCTTCGGGTAGCAACTGTC	AAATCCTCGGGAGAGTCTCTG
CXCL10(H)	GTGGCATTCAAGGAGTACCTC	TGATGGCCTTCGATTCTGGATT
CREB1(H)	ATTCACAGGAGTCAGTGGATAGT	CACCGTTACAGTGGTGATGG
